# CD133/CD49a discriminate between human pluripotent stem cell-derived pancreatic beta and alpha cells

**DOI:** 10.1016/j.stemcr.2026.102828

**Published:** 2026-03-05

**Authors:** Chenglei Tian, Yilin Di, Aisha Muhammad, Henrik Semb

**Affiliations:** 1 Institute of Translational Stem Cell Research, Helmholtz Diabetes Center, Helmholtz Zentrum Munchen, Munich, Germany; 2 School of Medicine and Health, Technical University of Munich, Munich, Germany

**Keywords:** human pluripotent stem cell, pancreas, stem cell-derived beta cells, stem cell-derived alpha cells, apical-basal polarity, beta cell purification

## Abstract

Human pluripotent stem cell (hPSC)-derived pancreatic beta cells provide an unlimited cell source for disease modeling and drug development. Generating highly purified beta cell populations from hPSCs remains challenging due to contamination by off-target and polyhormonal cells. Here, we present a robust cell-sorting-based purification strategy to enhance stem cell-derived beta (SC-beta) cell purity. Building on our previous work, we identified CD133 (PROM1) as a beta cell-enriched surface marker capable of distinguishing SC-beta cells from SC-alpha cells. Combining CD133 with the pan-endocrine marker CD49a (ITGA1) significantly increased beta cell enrichment while drastically reducing the fractions of alpha cells, polyhormonal cells, and ductal cells. This effect was consistent across multiple hPSC lines and differentiation protocols. Our approach yields SC-beta cell preparations with markedly improved purity, thereby advancing their application in disease modeling and drug development.

## Highlights

• CD133 is an SC-beta cell-enriched marker distinguishing SC-beta from SC-alpha cells
• Antibodies against CD133 and CD49a enrich SC-beta cells and reduce other cell types
• The sorting strategy is consistent across hPSC lines and differentiation protocols

## Teaser

In this article, Chenglei Tian and colleagues identified CD133 as an SC-beta cell-enriched surface marker capable of distinguishing SC-beta cells from SC-alpha cells. Combining cell sorting with antibodies against CD133 and the pan-endocrine marker CD49a significantly increased SC-beta cell enrichment while reducing the fractions of other cell populations, consistently across multiple hPSC lines and differentiation protocols.

## Introduction

Stem cell (SC)-derived pancreatic beta cells provide a renewable and scalable cell source for physiologically relevant disease models of diabetes, enabling the study of pathogenic mechanisms in a human context (Cujba et al., 2022; Hermann et al., 2023; Li et al., 2024; Singh et al., 2025). Moreover, these cells offer a powerful platform for drug discovery and development, facilitating the screening and evaluation of therapeutic candidates (Hu et al., 2025).

Over the past decade, stepwise differentiation protocols have enabled the generation of SC-beta cells (Balboa et al., 2022; Du et al., 2022; Rezania et al., 2014; Russ et al., 2015; Velazco-Cruz et al., 2019). However, achieving high purity in SC-beta cell preparations remains a significant challenge. The by-product populations, particularly alpha cells and polyhormonal cells (eventually will become alpha cells; Augsornworawat et al., 2023; Hiyoshi et al., 2024; Hiyoshi et al., 2022; Nair et al., 2019), can alter beta cell function through intercellular interactions, thereby limiting the use of SC-beta cells in disease modeling and drug development.

To address these concerns, efforts have been made to enrich beta cells using insulin reporter lines and antibody-based sorting strategies (Docherty et al., 2021; Nair et al., 2019; Parent et al., 2022; Saunders et al., 2019; Veres et al., 2019). Reporter-based approaches are highly effective but depend on genetic modification, which limits their translational potential. Antibody-based methods provide a more clinically relevant alternative; however, many rely on lab-generated reagents that are not commercially accessible and require additional validation (Docherty et al., 2021; Parent et al., 2022; Saunders et al., 2019). CD49a (ITGA1), identified through single-cell RNA sequencing, has recently been proposed as a commercially available marker for beta cell enrichment (Veres et al., 2019). Nevertheless, its specificity remains suboptimal (Molakandov et al., 2021), restricting its utility to broader endocrine enrichment (including alpha cells and polyhormonal cells) rather than selective beta cell purification. In addition, ENTPD3 can be used to enrich SC-beta cells, but it primarily marks a more mature and functional population (Docherty et al., 2021). Moreover, CD99 enriches human adult beta cells (Martens et al., 2018), and CD71 enriches mouse adult beta cells (Berthault et al., 2020), but neither marker has been validated in SC-beta cells.

Recent work has highlighted apical-basal polarity as a critical determinant of islet lineage specification (Tiemann et al., 2025; Tyler, 2003). As EPs mature, beta cells temporarily retain a diminished apical domain, while alpha cells remain non-polarized (Lof-Ohlin et al., 2017; Nyeng et al., 2019). Prominin-1 (PROM1, CD133), a transmembrane glycoprotein localized at apical membranes, has been identified as a marker of polarity, raising the possibility that it could distinguish immature beta cells from alpha and polyhormonal cells (Corbeil et al., 2010; Tiemann et al., 2025).

Here, we present a cell sorting-based strategy that combines CD133 and CD49a antibodies to enrich SC-beta cells. This dual-marker approach achieves more than 70% purity while eliminating off-target populations and alpha cells.

## Results

### CD133 is a beta cell-enriched cell surface marker in SC-islets

**Figure 1:**
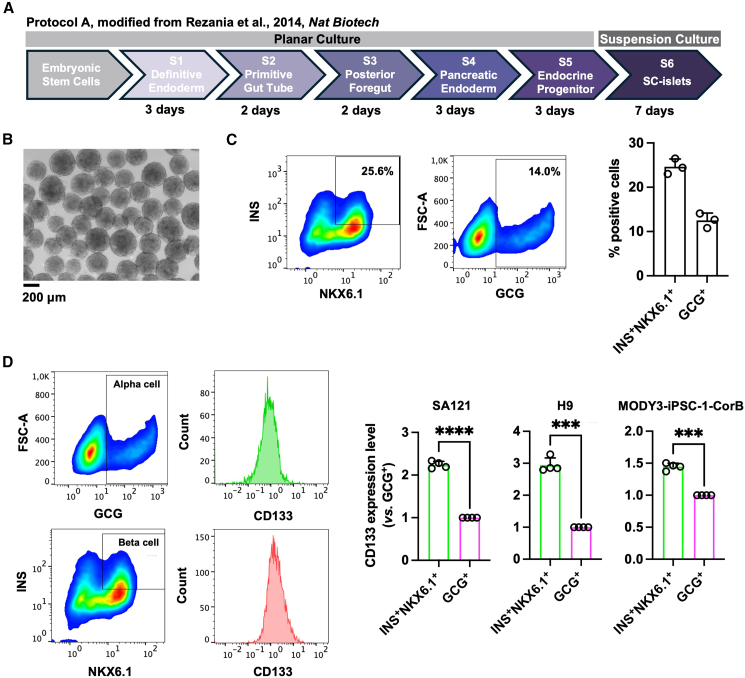
CD133 is a beta cell-enriched surface marker within SC-islets (A) Schematic diagram of a stepwise differentiation protocol to generate SC-islets from hPSCs. The protocol is modified from Rezania et al. *Nat Biotech* (Rezania et al., 2014). (B) Representative bright-field morphology images of SC-islets at the end of stage 6. Scale bars, 200 μm. Image is from the SA121 cell line. (C) Representative flow cytometry plots (left) and the quantification (right) of INSULIN (INS)^+^NKX6.1^+^ beta cells and Glucagon (GCG)^+^ alpha/polyhormonal cells in SC-islets at the end of differentiation. Data are presented as the mean ± SD (*n* = 3). Data are from SA121 (*n* = 1), H9 (*n* = 1), and MODY3-iPSC-1-CorB (*n* = 1) cell lines. (D) Representative flow cytometry plots (left) and the quantification (right) of CD133 expression between alpha/polyhormonal and beta cells at the end of differentiation. CD133 expression level is normalized by GCG^+^ cells. Data are presented as the mean ± SD (*n* = 4). ^∗∗∗^*p* < 0.001; ^∗∗∗∗^*p* < 0.0001. Data are from SA121, H9, and MODY3-iPSC-1-CorB cell lines.

Within SC-islets, alpha cells and beta cells are the predominant endocrine populations. We investigated whether CD133, a polarity-associated surface protein, could distinguish these populations. Using a modified Rezania et al. protocol (Rezania et al., 2014) (protocol A; Figure 1A), three genetically distinct human pluripotent stem cell (hPSC) lines (SA121, H9, and MODY3-iPSC-1-CorB; Figures S1A–S1C) generated comparable alpha/polyhormonal (marked by GCG^+^) and beta cell (marked INS^+^NKX6.1^+^) differentiation efficiencies at day 7 of stage 6 (Figures 1B, 1C, and S2). Across all lines, CD133 expression was consistently and significantly higher in beta cells than in alpha/polyhormonal cells, identifying CD133 as a beta cell-enriched marker (Figure 1D).

In addition, CD133 expression in beta cells decreased during *in vitro* maturation (Figures S3A and S3B), consistent with reports showing lower CD133 (encoded by *PROM1*) levels in *in vivo*-grafted (mature) versus *in vitro*-derived (immature) SC-beta cells (Augsornworawat et al., 2020), suggesting that CD133 marks newly formed, immature beta cells.

### CD133-based sorting enriches beta and ductal-like cells

**Figure 2:**
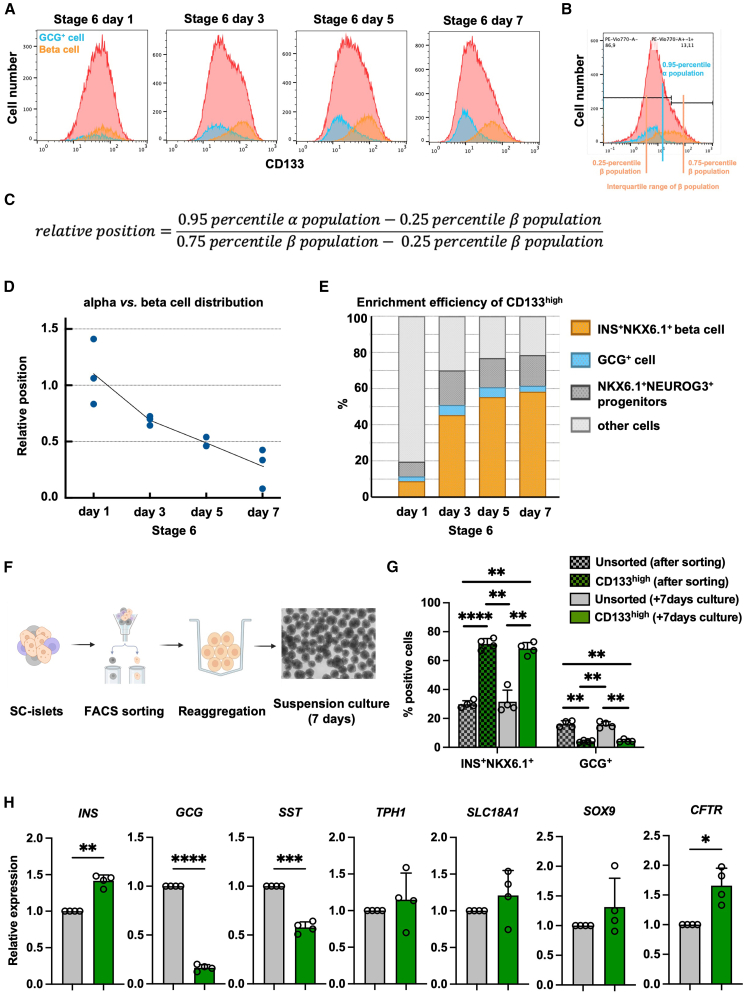
CD133 as a cell surface marker for excluding glucagon-producing cells and enhancing the purity of SC-derived beta cells (A) Time course of GCG^+^ alpha/polyhormonal cells (blue) and INS^+^NKX6.1^+^ beta cells (orange) distribution among all cell populations (red) during stage 6 differentiation. NEUROG3^+^ cells, representing the overall endocrine population, were identified as the red histogram. Within this population, GCG^+^ cells were depicted in blue, and INS^+^NKX6.1^+^ cells in orange. All population histograms were plotted according to CD133 intensity. Overlaying these subpopulations on the total endocrine population enabled visualization of lineage segregation based on CD133 expression, with reduced overlap indicating clearer distinction between alpha and beta lineages. Data are from the NEUROG3-GFP cell line. (B) Illustration of the interquartile range of beta cells and the 0.95 percentile of alpha/polyhormonal cells. (C) Algorithm to define the relative position index. Values approaching 1 indicated substantial overlap, while values approaching 0 reflected distinct separation. (D) Alpha cell vs. beta cell relative position index based on CD133 expression level along stage 6 differentiation. Data are from the NEUROG3-GFP cell line. (E) Flow cytometry analysis of GCG^+^ alpha/polyhormonal cells, INS^+^NKX6.1^+^ beta cells, NKX6.1^+^NEUROG3^+^ progenitors, and other cells based on gating selection during stage 6 differentiation. Data are from the NEUROG3-GFP cell line. (F) Schematic diagram of the cell sorting, reaggregation, and subsequent suspension culture. At the end of differentiation, cells were sorted based on CD133 expression. The sorted cells were then reaggregated, and one day after reaggregation, they were transferred to a 6-well suspension culture plate for 7 days of culture. The cells were collected after sorting and after 7 days of culture for analysis. (G) Flow cytometry analysis of INS^+^NKX6.1^+^ beta cells and GCG^+^ alpha/polyhormonal cells in unsorted and CD133-sorted populations. Data are presented as the mean ± SD (*n* = 4). ^∗∗^*p* < 0.01; ^∗∗∗∗^*p* < 0.0001. Data are from the SA121 cell line. (H) RT-qPCR assessment of marker genes in unsorted and CD133-sorted populations. Data are presented as the mean ± SD (*n* = 4). ^∗^*p* < 0.05; ^∗∗^*p* < 0.01; ^∗∗∗^*p* < 0.001; ^∗∗∗∗^*p* < 0.0001. Data are from the SA121 cell line.

Flow cytometry analysis integrated alpha/polyhormonal and beta subpopulations according to CD133 expression intensity, enabling clear visualization of their temporal dynamics (Figure 2A). As the EP stage represents a dynamically shifting microenvironment where progenitors continually alter their polarity status (Tiemann et al., 2025), the timing of cell sorting is critical for achieving high enrichment efficiency. To strengthen the analysis, the 0.95 percentile of the alpha/polyhormonal population was used as the threshold to define its upper boundary, reflecting the aim of excluding this population. In contrast, the interquartile range of the beta population was applied to characterize the distribution of beta cells (Figure 2B). Based on the algorithm (Figure 2C), a higher relative position index indicates greater overlap between the two populations, whereas values closer to 0 reflect minimal overlap. During stage 6 differentiation, alpha/polyhormonal and beta cell populations became progressively more distinct (Figure 2D). By day 7 of stage 6, a clear separation was observed, with the NEUROG3^+^CD133^high^ population representing a substantial fraction of beta cells (Figure 2E). These findings suggest that adjusting sorting to this window could yield optimal enrichment efficiency.

To evaluate the utility of CD133 for beta cell purification, stage 6 day 7 SC-islets were sorted into CD133^high^ and unsorted populations, reaggregated, and maintained in suspension culture for 7 days (Figure 2F). Our results showed that CD133 expression was higher in beta cells than in GCG^+^ alpha/polyhormonal cells (Figure 1D). As our differentiation protocol typically yields 20%–30% beta cells (Figure 1C), we set the CD133^high^ sorting threshold at 20% to efficiently and consistently enrich beta cells (Figure S4A). Flow cytometry revealed that the CD133^high^ sorted cells contained a significantly higher proportion of INS^+^NKX6.1^+^ beta cells and a markedly reduced proportion of GCG^+^ alpha/polyhormonal cells compared with unsorted controls (Figure 2G). In addition, because most progenitor fates are determined by the end of stage 6, the proportions of INS^+^NKX6.1^+^ beta cells and GCG^+^ alpha cells remained stable following re-aggregation and 7 days of suspension culture (Figure 2G). Consistent with these findings, quantitative real-time PCR (RT-qPCR) analysis showed elevated INS expression and reduced GCG and SST transcripts in CD133^high^ cells (Figure 2H), indicating effective enrichment of beta cells and depletion of other types of endocrine cells.

However, CD133^high^ sorting also led to enrichment of CFTR-expressing cells, a marker of ductal-like cells, as well as modest increases in SOX9, another ductal progenitor marker (Figure 2H). This is consistent with the known fact that ductal cells, like newborn beta cells, are polarized. These results suggest that while CD133 is effective for beta cell enrichment and the removal of alpha and polyhormonal cells, additional markers are required to exclude ductal contaminants from the purified population.

### Combining CD133 with CD49a eliminates ductal contaminants, thereby increasing beta cell purity

CD49a (ITGA1) is a pan-endocrine cell surface marker that is broadly expressed in endocrine cells but absent from ductal epithelium (Veres et al., 2019), making it a suitable candidate for excluding CD133^+^ ductal contaminants.

**Figure 3:**
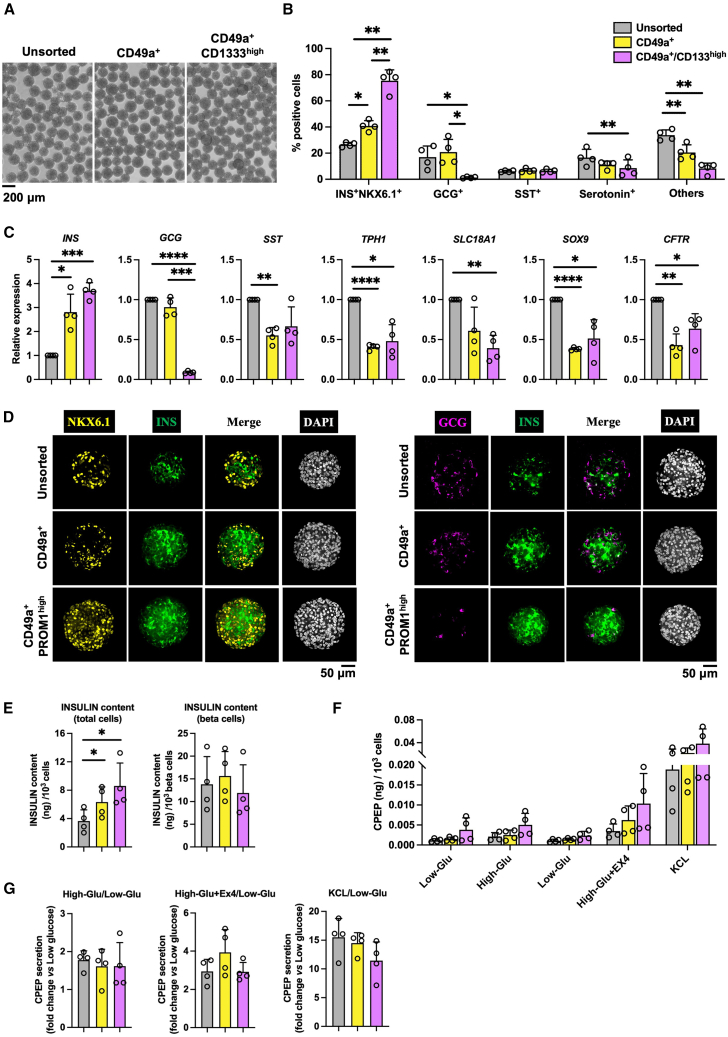
Enhancing the purity of SC-derived beta cells through CD133 and CD49a-based sorting (A) Representative bright-field images of unsorted, CD49a-sorted, and CD49a/CD133-sorted cells after 7 days of reaggregation. Scale bars, 200 μm. (B) Flow cytometry analysis of INS^+^NKX6.1^+^ beta cells, GCG^+^ alpha/polyhormonal cells, Somatostatin (SST)^+^ delta cells, Serotonin^+^ enterochromaffin-like cells, and other cells in unsorted, CD49a-sorted and CD49a/CD133-sorted populations. Staining panels included INS + NKX6.1, GCG + SST, and Serotonin. “Other cells” represent all cells excluding INS^+^NKX6.1^+^, GCG^+^, SST^+^, and Serotonin^+^ populations. The GCG^+^ population consists of GCG single-positive alpha cells and GCG^+^INS^+^ polyhormonal cells (lack NKX6.1 expression (Peterson et al., 2020; Veres et al., 2019). Data are presented as the mean ± SD (*n* = 4). ^∗^*p* < 0.05; ^∗∗^*p* < 0.01. (C) RT-qPCR assessment of marker genes in unsorted, CD49a sorted, and CD49a/CD133 sorted populations. Data are presented as the mean ± SD (*n* = 4). ^∗^*p* < 0.05; ^∗∗^*p* < 0.01; ^∗∗∗^*p* < 0.001; ^∗∗∗∗^*p* < 0.0001. (D) Immunostaining on INS, NKX6.1, and GCG expression in unsorted, CD49a-sorted, and CD49a/CD133-sorted populations. Scale bars, 50 μm. (E) Insulin content measured by ELISA in unsorted, CD49a-sorted, and CD49a/CD133-sorted cell populations. Left: insulin content normalized by 10^3^ total cells; right: insulin content normalized by 10^3^ beta cells (total cells/the percentage of beta cells). Data are presented as the mean ± SD (*n* = 4). ^∗^*p* < 0.05; ^∗∗^*p* < 0.01. (F) The C-peptide secretion level after low glucose (Low-Glu), high glucose (Hi-Glu), Low-Glu, high glucose plus Exendin-4 (Hi-Glu+Ex4), and low glucose plus KCl (KCl) treatment. Data are presented as mean ± SD (*n* = 4). (G) C-peptide secretion stimulation index in static GSIS measured by ELISA in unsorted, CD49a-sorted, and CD49a/CD133-sorted cell populations. Data are presented as the mean ± SD (*n* = 4). All the data are from the SA121 cell line.

Because the endocrine cell composition remained stable for 7 days after re-aggregation (Figure 2B), only cells at this time point were analyzed. Compared with unsorted and CD49a alone-sorted cells, dual CD49a/CD133 selection yielded the highest beta cell purity (>75% INS^+^NKX6.1^+^) and most efficient removal of GCG^+^ alpha and polyhormonal cells (Figures 3B and 3C). In addition, CD49a/CD133-sorted cells show a significant reduction of enterochromaffin-like cells compared with unsorted cells (Figures 3B and 3C). Importantly, ductal markers *CFTR* and *SOX9* were markedly reduced in the CD49a alone and dual CD49a/CD133-sorted population, confirming effective removal of ductal-like contaminants (Figure 3C). Immunofluorescence analysis corroborated these results, showing a predominance of INS^+^NKX6.1^+^ beta cells and near-complete absence of GCG^+^ alpha and polyhormonal cells in the dual CD49a/CD133-sorted population (Figure 3D).

Additionally, the higher beta cell proportion in dual CD49a/CD133-sorted populations resulted in an overall higher total insulin content compared with unsorted or CD49a-only sorted cells, but did not increase the insulin content per beta cell (Figure 3E). These cells secreted C-peptide in response to high glucose and exendin-4 stimulation (Figure 3F). However, the glucose stimulation index did not differ significantly among unsorted, CD49a-only, and dual-sorted groups, indicating that CD49a and/or CD133 selection enriches beta cell number but does not distinguish between functionally mature and less mature beta cells.

### Dual CD49a/CD133 sorting reproducibly enriches beta cells across hPSC lines and differentiation protocols

To assess the robustness of the dual-marker sorting strategy, we used the H9 hESC and MODY3-iPSC-1-CorB lines differentiated under protocol A (Rezania et al., 2014) (Figures S5 and S6). In each setting, dual CD49a/CD133 sorting consistently enriched beta cells to >70% purity, minimized alpha/polyhormonal, enterochromaffin-like, and ductal-like cells, and improved total insulin content (Figures S5A–S5D and S6A–S6D). The glucose stimulation index remained comparable across groups, consistent with results from the SA121 line (Figures S5E and S6E).

The reproducibility of this approach was further tested by applying CD49a/CD133 sorting to SC-islets generated using an alternative differentiation protocol (protocol B, modified from Du et al., 2022; Figures S7A and S7B). As observed with protocol A, dual CD49a/CD133 sorting markedly increased the proportion of INS^+^NKX6.1^+^ beta cells while reducing alpha/polyhormonal, enterochromaffin-like, and ductal-like cell contaminants (Figure S7C). Transcript analysis confirmed elevated *INS* expression with reduced *GCG*, *TPH1*, *SLC18A1*, *SOX9*, and *CFTR* levels in dual-sorted populations (Figure S7D), accompanied by increased insulin content (Figure S7E) and proper GSIS performance (Figure S7F). In addition, testing CD133 antibody dilutions from 1:5 to 1:20 showed comparable beta cell sorting efficiencies (Figure S8).

Together, these findings demonstrate that CD49a/CD133 dual-marker selection is a robust and generalizable strategy for beta cell enrichment, effective across multiple hPSC lines, and differentiation protocols.

## Discussion

In this study, we establish a new cell sorting strategy for the purification of SC-beta cells. Compared with existing methods, our approach provides three main advantages: (1) more effective enrichment of SC-beta cells from the endocrine pool with efficient exclusion of GCG^+^ alpha/polyhormonal cells; (2) reliance on cell surface markers compatible with clinical manufacturing workflows, supporting good manufacturing practice (GMP)-compliant implementation; and (3) use of commercially available, well-validated antibodies, thereby enhancing reproducibility and translational feasibility. Together, these advances markedly improve SC-beta cell purity and strengthen their potential for safe and effective application in disease modeling and drug development.

Previous studies have highlighted a critical role for apical-basal polarity in endocrine lineage specification (Tiemann et al., 2025). During mouse pancreatic development, alpha cells are predominantly generated at early stages when the epithelium remains largely non-polarized, whereas beta cells emerge preferentially at later stages, coinciding with the establishment of epithelial polarity and lumen formation (Johansson et al., 2007; Nyeng et al., 2019; Tiemann et al., 2025). Furthermore, live-cell imaging studies revealed that newly formed beta cells transiently retain apical polarity within the epithelium and after delamination (Nyeng et al., 2019). This provided the rationale for employing the apically expressed CD133, an epithelial progenitor marker that is not expressed in mature hormone-positive endocrine cells (Sugiyama et al., 2007), as a surface marker to isolate SC-beta cells.

Still, CD133 alone is not sufficient for the purification of SC-beta cells from a heterogeneous cell population, because it will also target other polarized cells, such as ductal cells (Figures 2G and 2H). To remove ductal cells, we decided to also use the pan-endocrine marker: CD49a, since it is absent from ductal cells (Gharibi et al., 2017; Veres et al., 2019). Based on these properties, we combined CD133 and CD49a to enrich for beta cells while minimizing contamination from ductal cells. To the best of our knowledge, CD133 is the only cell surface marker shown to efficiently enrich immature beta cells, such as SC-beta cells, from the endocrine populations. The fact that CD133, a polarity-associated marker, can distinguish SC-beta cells from alpha/polyhormonal cells further suggests that additional polarity-linked surface markers may be exploited for SC-beta cell purification.

While this study demonstrates that the CD133/CD49a double-sorting strategy provides a robust approach for enriching SC-beta cells, several limitations should be acknowledged. First, this strategy does not achieve complete beta-cell purity, with residual non-beta endocrine cells and progenitor populations remaining, which may raise safety considerations in therapeutic settings and warrant further evaluation in animal transplantation models. Second, it remains to be determined whether the performance of this purification strategy will differ when applied to more efficient beta cell differentiation platforms.

## Methods

### Flow cytometry sorting and analysis

Cells were dissociated by Accutase for 20 min at 37°C (10 mL Accutase per 6-well suspension plate). The dissociation was terminated by adding an equal volume of stage 6 basal medium, and the cell suspension was passed through a 40 μm strainer to obtain single cells. The cells were then counted, centrifuged, and resuspended in MACSQuant Tyto Running Buffer (Miltenyi Biotec, #130-107-207) supplemented with CD133 antibody (1:10) and CD49a antibody (1:50) (100 μL buffer per 1 × 10^6^ cells), and incubated for 15 min at room temperature. Subsequently, the cells were washed once with the Running Buffer and sorted on a Miltenyi MACSQuant Tyto sorter using gates to isolate single cells based on scatter characteristics. For CD133^high^ sorting, the gate was set to include the top 20% of high-positive cells; for CD49a, gating was based on the clear separation from the negative population.

### Quantification and statistical analysis

Statistical analyses were performed with GraphPad Prism 10 (GraphPad Software). Unless otherwise noted, a paired nonparametric test (Wilcoxon matched-pairs signed-rank test) was used to assess significance. An unpaired nonparametric test was used for unpaired data (Mann-Whitney test). Asterisks denote *p* values as follows: ^∗^*p* < 0.05; ^∗∗^*p* < 0.01; ^∗∗∗^*p* < 0.001; ^∗∗∗∗^*p* < 0.0001. Unless otherwise noted, each *N* represents a biological replicate (one independent differentiation experiment). Data figures illustrate the mean with standard deviation (SD) and the values of individual biological replicates.

## Resource availability

### Lead contact

Further information and requests for resources and reagents should be directed to and will be fulfilled by the lead contact, Henrik Semb (henrik.semb@helmholtz-munich.de).

### Materials availability

All unique/stable reagents in this study are available from the lead contact with a completed Materials Transfer Agreement.

### Data and code availability

This study did not generate any custom code. All analyses were performed using standard commercial and open-source software, as detailed in the methods section. All the data collected in this study are available from the lead contact upon a reasonable request.

## Acknowledgments

All the work is supported by the European Union’s Horizon 2020 research and innovation program (ISLET, no. 874839); the 10.13039/501100002347Federal Ministry of Education and Research (BMBF) project (eISLET, no. 031L0251); and the 10.13039/501100013295Helmholtz Zentrum München.

## Author contributions

C.T., Y.D., and H.S. designed the experiments; C.T., Y.D., and A.M. performed the experiments; C.T. and Y.D. performed analyses; C.T. and H.S. wrote the manuscript.

## Declaration of interests

C.T. and H.S. are listed as inventors on an international patent application partially based on this work.

## Supplemental information

Document S1. Figures S1–S8 and Table S1

Document S2. Article plus supplemental information
