# Supplementary material for: CD133/CD49a discriminate between human pluripotent stem cell-derived pancreatic beta and alpha cells
Source: Stem Cell Reports. 2026 Mar 5;21(4):102828. doi: 10.1016/j.stemcr.2026.102828 (PMC13083789; doi:10.1016/j.stemcr.2026.102828)
Supplement: Document S1. Figures S1–S8 and Table S1 [file mmc1.pdf]

Stem Cell Reports, Volume *21*

Supplemental Information

CD133/CD49a discriminate between human pluripotent stem cell-derived pancreatic beta and alpha cells

Chenglei Tian, Yilin Di, Aisha Muhammad, and Henrik Semb

Supplementary Information

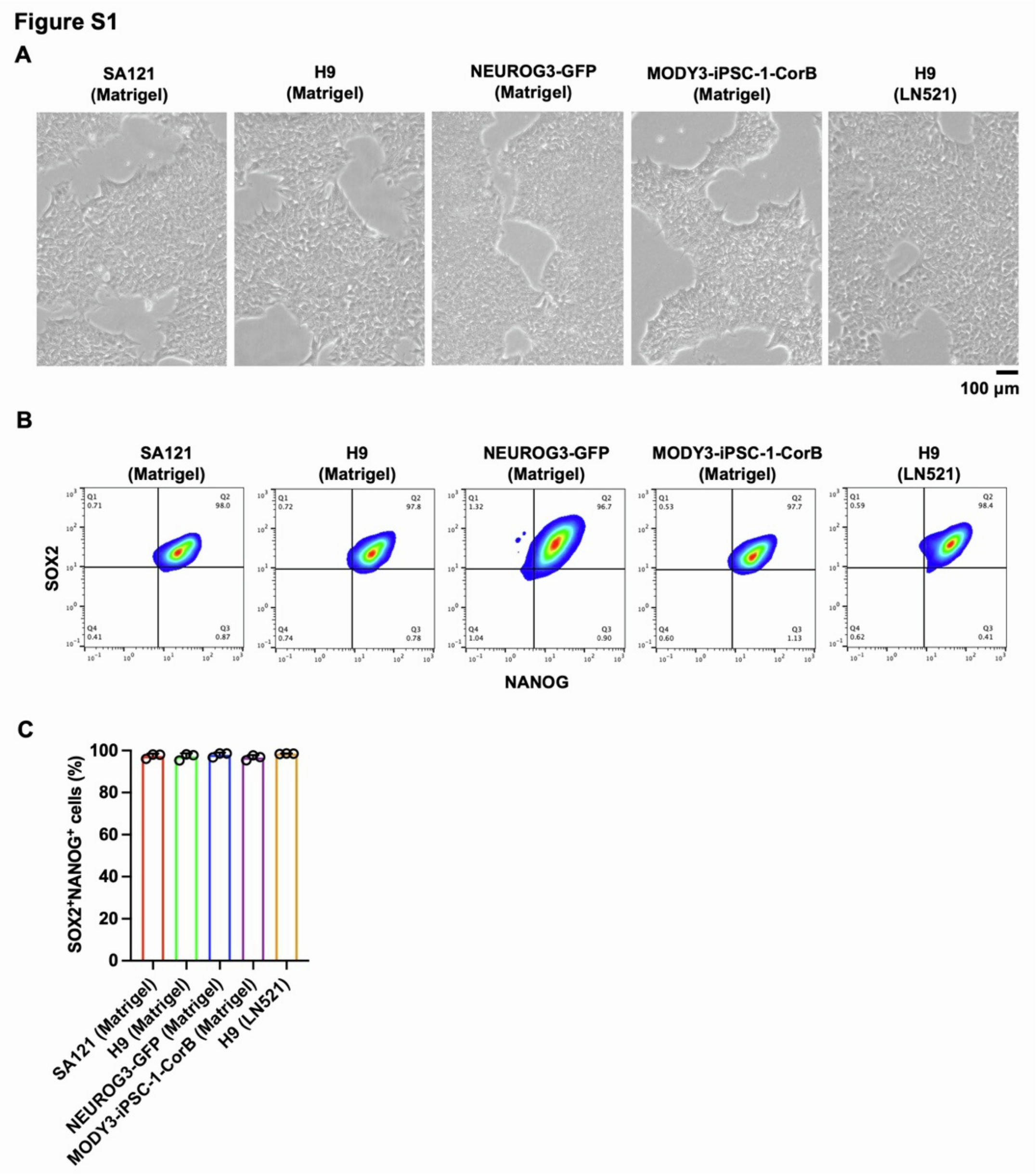

Figure S1. Culture and pluripotency characterization of human pluripotent stem cells (hPSCs), related to Figures 1-3.

(A) Representative bright-field images of Matrigel cultured SA121 human embryonic stem cells (hESCs), H9 hESCs, NEUROG3-GFP hESCs, MODY3-iPSC-1-CorB human induced pluripotent stem cells (hiPSCs), and Laminin 521 (LN521) cultured H9 hESCs. Scale bar, 100 μm.

(B) Representative flow cytometry plots of pluripotent markers (SOX2 and NANOG) in the hPSCs.

(C) The quantification of pluripotent markers (SOX2 and NANOG) in the hPSCs. Data are presented as the mean ± SD (n = 3).

Data are from SA121, H9, NEUROG3-GFP and MODY3-iPSC-1-CorB cell lines.

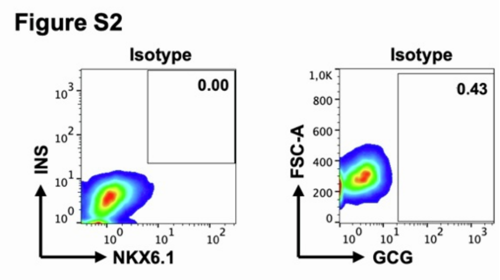

Figure S2. Representative flow cytometry plots of the isotype control for alpha and beta cell gating, related to Figure 1.

Representative flow cytometry plots of the isotype control for Figure 1C. Data are from the SA121 cell line.

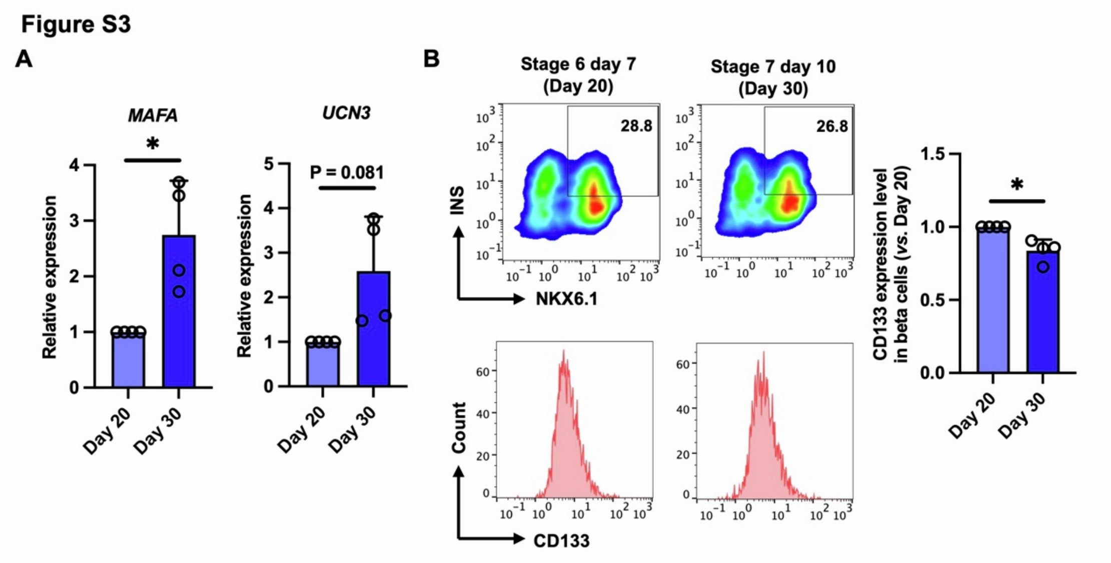

Figure S3. CD133 expression in SC-beta cells during *in vitro* maturation, related to Figure 1.

(A) RT-qPCR assessment of maturation-related genes (*MAFA* and *UCN3*) in Day 20 and Day 30 SC-islets. Data are presented as the mean ± SD (n = 4). *$p < 0.05$.

(B) Representative flow cytometry plots (left) and the quantification (right) of CD133 expression in beta cells before and after *in vitro* maturation. CD133 levels are normalized to Day 20 beta cells. Data are from the SA121 cell line. Data are presented as the mean ± SD (n = 4). *$p < 0.05$.

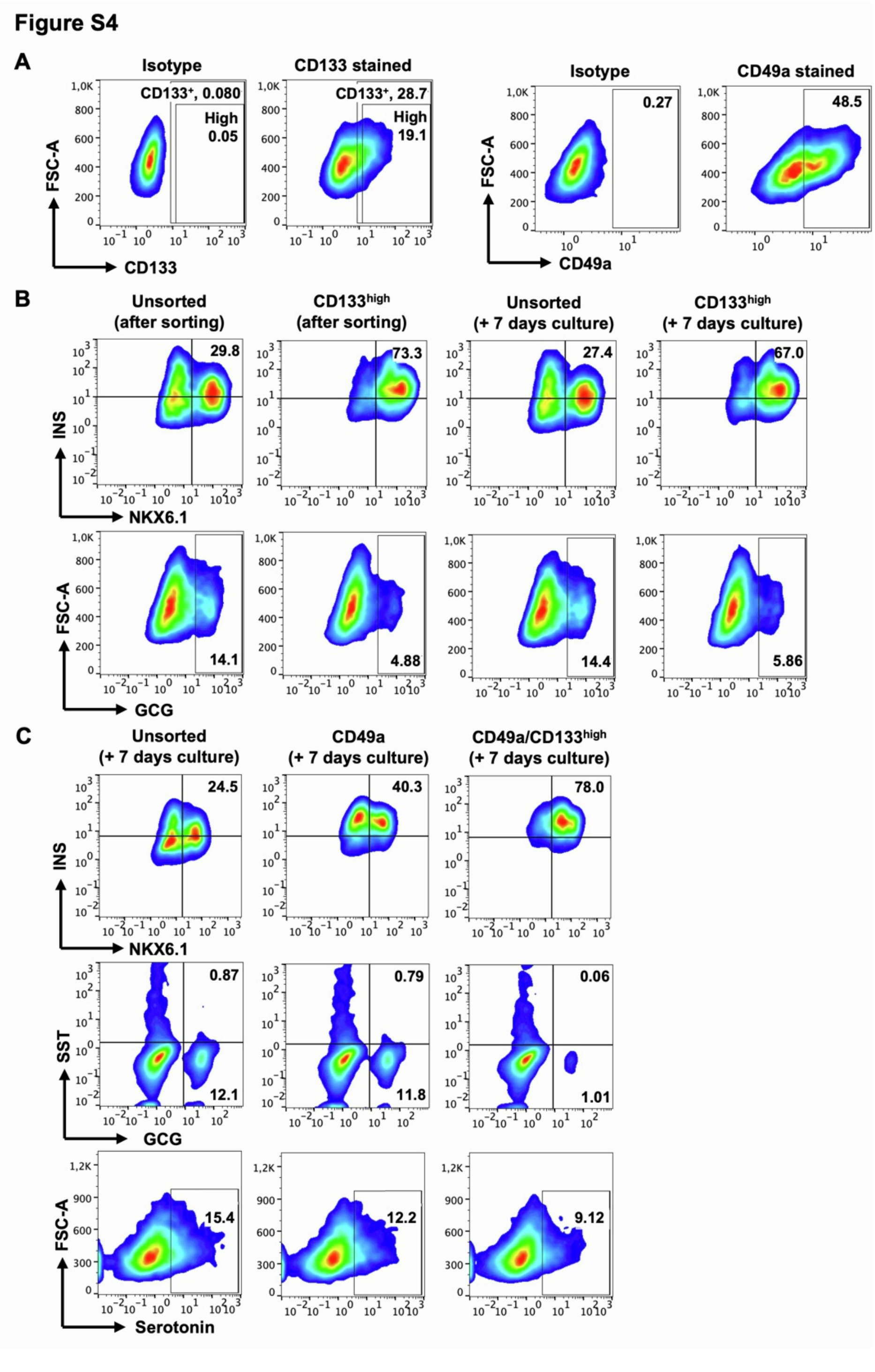

Figure S4. Flow cytometry analysis of purified SC-beta cells, related to Figures 2 and 3.

(A) Representative flow cytometry plots of isotype control, CD133 and CD49a staining of Day 20 samples.

(B) Representative flow cytometry plots of $INS^+NKX6.1^+$ beta cells and GCG^+ alpha/polyhormonal cells in unsorted and $CD133^{high}$ populations after sorting and after 7 days of culture.

(C) Representative flow cytometry plots of INS$^+$NKX6.1$^+$ beta cells, GCG$^+$ alpha/polyhormonal cells, SST$^+$ delta cells and Serotonin$^+$ enterochromaffin cells in unsorted, CD49a$^+$ and CD49a$^+$/CD133^{high} populations after 7 days of culture.

Data are from the SA121 cell line.

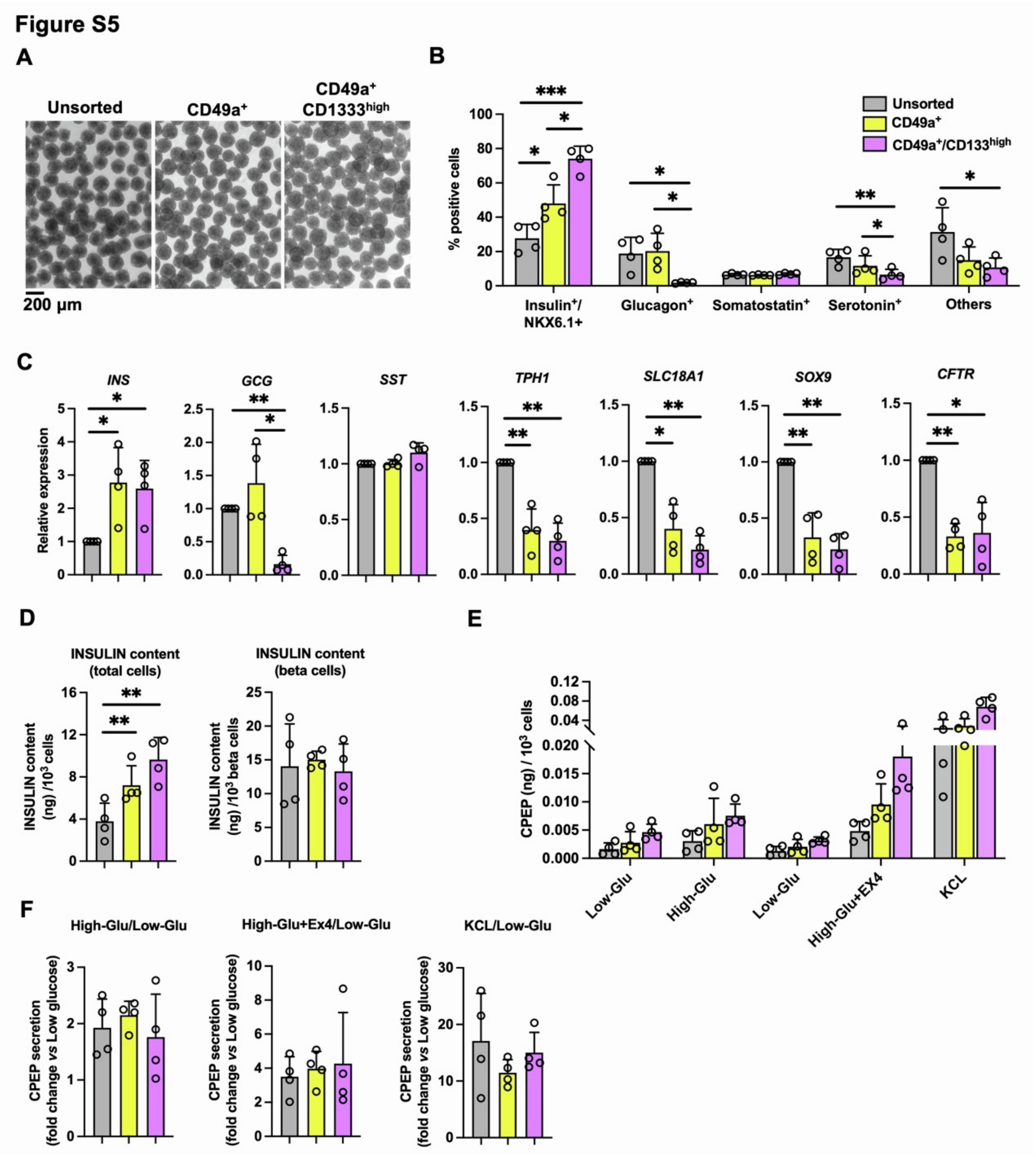

Figure S5. Enhancing the purity of H9 hESC-derived beta cells through CD133 and CD49a-based sorting, related to Figure 3.

(A) Representative bright-field images of unsorted, CD49a-sorted, and CD49a/CD133-sorted cells after 7 days of reaggregation. Scale bar, 200 µm.

(B) Flow cytometry analysis of $INS^{+}NKX6.1^{+}$ beta cells, GCG^{+} alpha/polyhormonal cells, Somatostatin $(SST)^{+}$ delta cells, $Serotonin^{+}$ enterochromaffin cells, and other cells in unsorted, CD49a-sorted and CD49a/CD133-sorted populations. Data are presented as the mean ± SD (n = 4). *$p < 0.05$; **$p < 0.01$; ***$p < 0.001$.

(C) RT-qPCR assessment of marker genes in unsorted, CD49a sorted and CD49a/CD133 sorted populations. Data are presented as the mean ± SD (n = 4). *$p < 0.05$; **$p < 0.01$.

(D) Insulin content measured by ELISA in unsorted, CD49a-sorted, and CD49a/CD133-sorted cell populations. Left: Insulin content normalized by 10^3 total cells; Right: Insulin content

normalized by 10^3 beta cells (total cells / the percentage of beta cells). Data are presented as the mean ± SD (n = 4). **$p < 0.01$.

(E) The C-peptide secretion level after Low glucose (Low-Glu), High glucose (Hi-Glu), Low-Glu, High glucose plus Exendin-4 (Hi-Glu+Ex4) and Low glucose plus KCl (KCl) treatment. Data are presented as mean ± SD (n = 4).

(F) C-peptide secretion stimulation index in static GSIS measured by ELISA in unsorted, CD49a-sorted, and CD49a/CD133-sorted cell populations. Data are presented as the mean ± SD (n = 4).

All the data are from the H9 cell line.

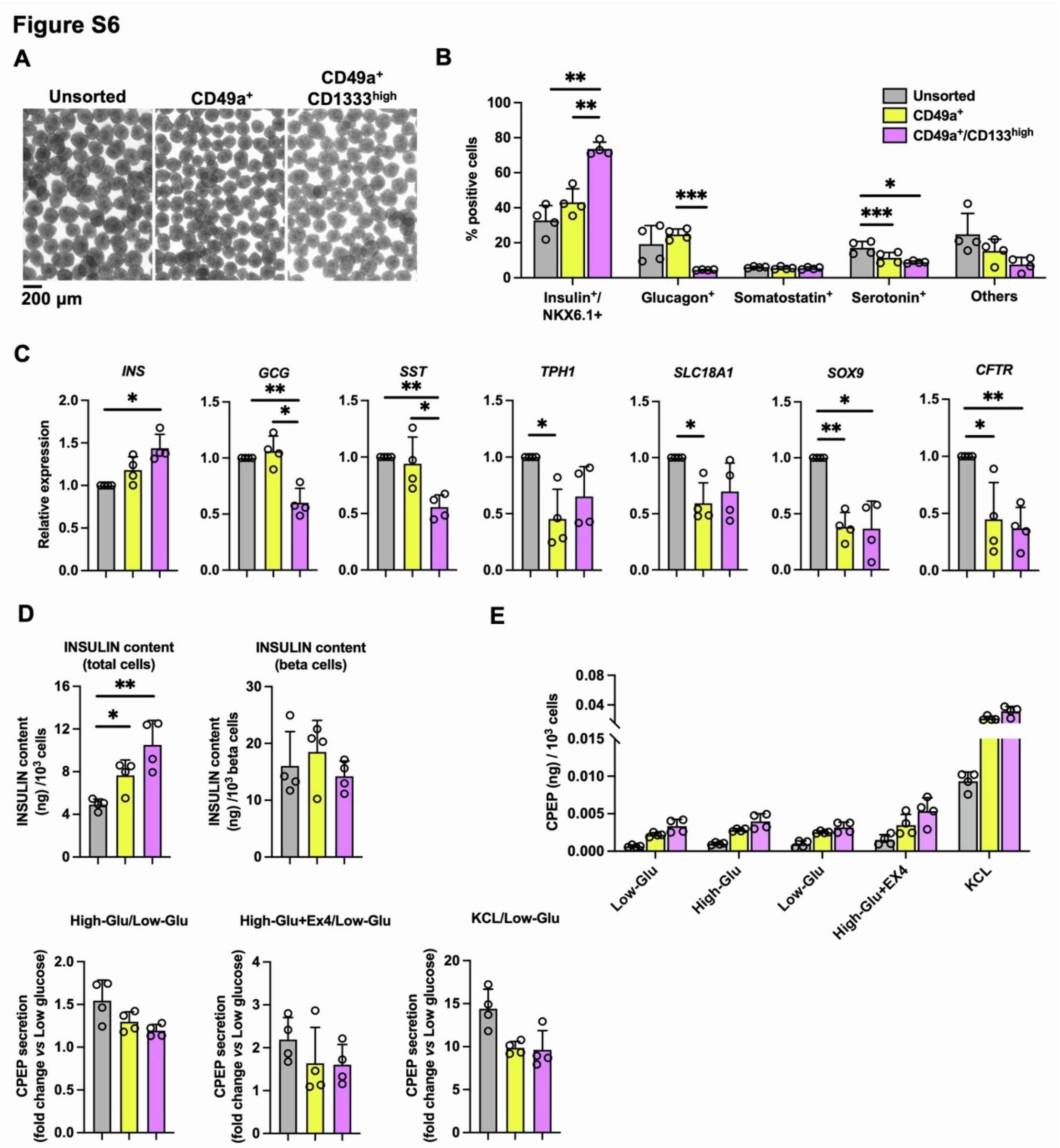

Figure S6. Enhancing the purity of MODY3-iPSC-1-CorB hESC-derived beta cells through CD133 and CD49a-based sorting, related to Figure 3.

(A) Representative bright-field images of unsorted, CD49a-sorted, and CD49a/CD133-sorted cells after 7 days of reaggregation. Scale bar, 200 µm.

(B) Flow cytometry analysis of $INS^{+}NKX6.1^{+}$ beta cells, GCG^{+} alpha/polyhormonal cells, Somatostatin $(SST)^{+}$ delta cells, $Serotonin^{+}$ enterochromaffin cells, and other cells in unsorted, CD49a-sorted and CD49a/CD133-sorted populations. Data are presented as the mean ± SD (n = 4). $*p < 0.05$; $**p < 0.01$; $***p < 0.001$.

(C) RT-qPCR assessment of marker genes in unsorted, CD49a sorted and CD49a/CD133 sorted populations. Data are presented as the mean ± SD (n = 4). $*p < 0.05$; $**p < 0.01$.

(D) Insulin content measured by ELISA in unsorted, CD49a-sorted, and CD49a/CD133-sorted cell populations. Left: Insulin content normalized by 10^3 total cells; Right: Insulin content

normalized by 10^3 beta cells (total cells / the percentage of beta cells). Data are presented as the mean ± SD (n = 4). *p < 0.05; **p < 0.01.

(E) The C-peptide secretion level after Low glucose (Low-Glu), High glucose (Hi-Glu), Low-Glu, High glucose plus Exendin-4 (Hi-Glu+Ex4) and Low glucose plus KCl (KCl) treatment. Data are presented as mean ± SD (n = 4).

(F) C-peptide secretion stimulation index in static GSIS measured by ELISA in unsorted, CD49a-sorted, and CD49a/CD133-sorted cell populations. Data are presented as the mean ± SD (n = 4).

All the data are from the MODY3-iPSC-1-CorB cell line.

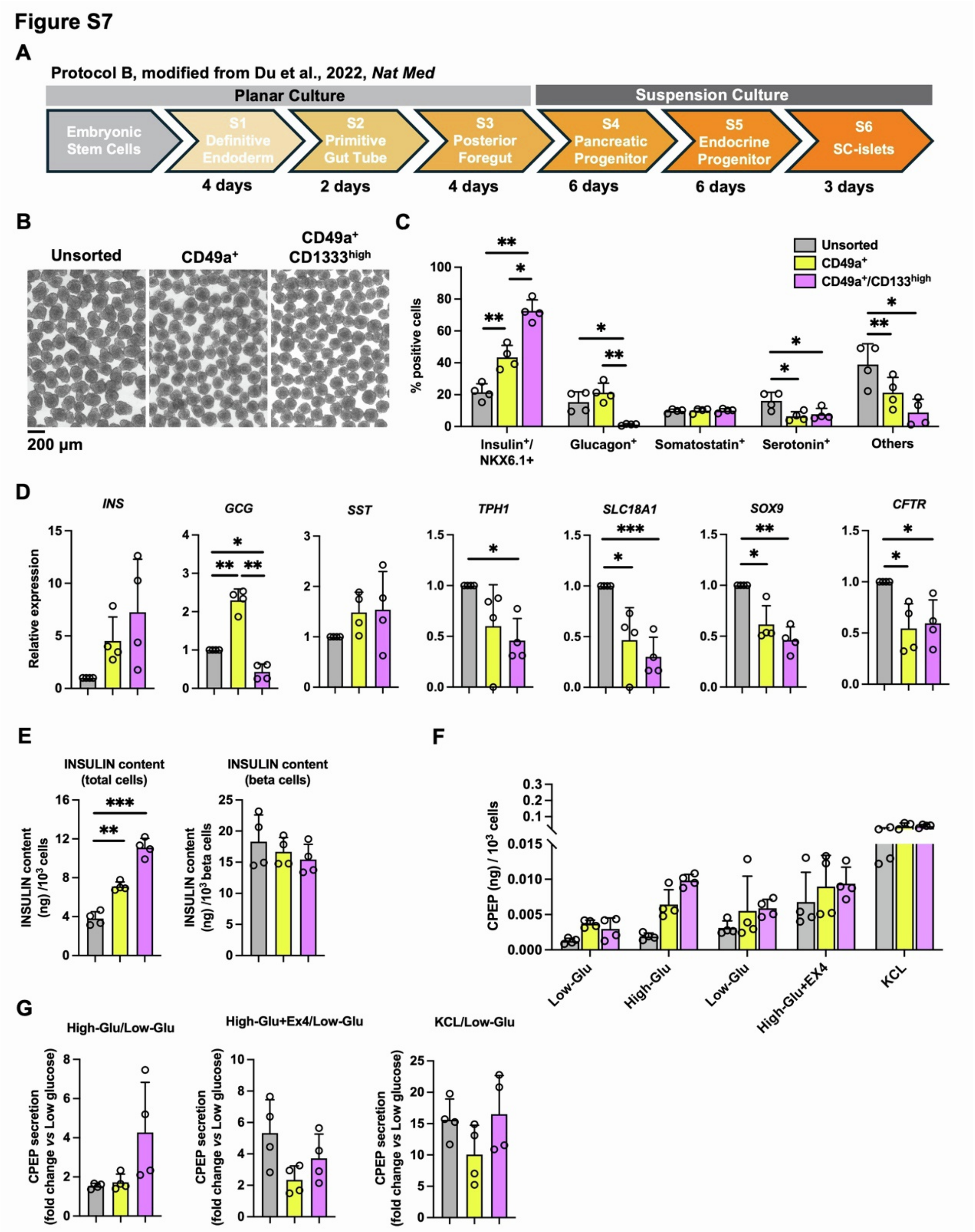

Figure S7. Enhancing the purity of SC-derived beta cells via CD133 and CD49a-based sorting across distinct differentiation protocols, related to Figure 3.

(A) Schematic diagram of a stepwise differentiation protocol to generate SC-islets from hESC. The protocol is modified from Du et al. *Nat Med* (Du et al., 2022).

(B) Representative bright-field images of unsorted, CD49a-sorted, and CD49a/CD133-sorted cells after 7 days of reaggregation. Scale bar, 200 μm.

(C) Flow cytometry analysis of $INS^{+}NKX6.1^{+}$ beta cells, GCG^{+} alpha/polyhormonal cells, Somatostatin $(SST)^{+}$ delta cells, $Serotonin^{+}$ enterochromaffin-like cells, and other cells in

unsorted, CD49a-sorted and CD49a/CD133-sorted populations. Data are presented as the mean ± SD (n = 4). *$p < 0.05$; **$p < 0.01$.

(D) RT-qPCR assessment of marker genes in unsorted, CD49a sorted and CD49a/CD133 sorted populations. Data are presented as the mean ± SD (n = 4). *$p < 0.05$; **$p < 0.01$; ***$p < 0.001$.

(E) Insulin content measured by ELISA in unsorted, CD49a-sorted, and CD49a/CD133-sorted cell populations. Left: Insulin content normalized by 10^3 total cells; Right: Insulin content normalized by 10^3 beta cells (total cells / the percentage of beta cells). Data are presented as the mean ± SD (n = 4). *$p < 0.05$; **$p < 0.01$; ***$p < 0.001$.

(F) The C-peptide secretion level after Low glucose (Low-Glu), High glucose (Hi-Glu), Low-Glu, High glucose plus Exendin-4 (Hi-Glu+Ex4) and Low glucose plus KCl (KCl) treatment. Data are presented as mean ± SD (n = 4).

(G) C-peptide secretion stimulation index in static GSIS measured by ELISA in unsorted, CD49a-sorted, and CD49a/CD133-sorted cell populations. Data are presented as the mean ± SD (n = 4).

All the data are from the H9 cell line.

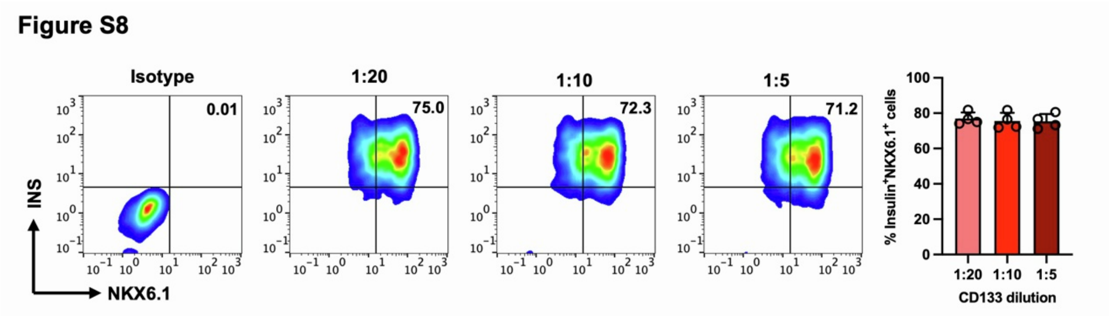

Figure S8. Dilutions of the CD133 antibody from 1:5 to 1:20 yielded comparable beta cell sorting efficiencies, related to Figure 3.

Representative flow cytometry plots (left) and analyses (right) of $INS^{+}NKX6.1^{+}$ beta cells within the $CD49a^{+}$ and $CD133^{high}$ populations using different CD133 antibody dilutions. Data are presented as mean ± SD (n = 4).

Supplemental Experimental Procedures

KEY RESOURCES TABLE

REAGENT or RESOURCE	SOURCE	IDENTIFIER
**Antibodies**		
PE anti-INSULIN	Cell Signaling	#8508<br>RRID: AB_11179076
Alexa fluor 647 anti-INSULIN	BD Biosciences	#565689<br>RRID: AB_2739331
Alexa fluor 488 anti-NKX6.1	BD Biosciences	#563338<br>RRID: AB_2738144
PE anti-NKX6.1	BD Biosciences	#563023<br>RRID: AB_2716792
PE anti-Glucagon	BD Biosciences	#565860<br>RRID: AB_2739382
BV421 anti-Glucagon	BD Biosciences	#565891<br>RRID: AB_2739385
Alexa fluor 647 anti-Somatostatin	BD Biosciences	#570384<br>RRID: AB_3685704
APC anti-Serotonin	Miltenyi Biotec	#130-132-489<br>RRID: AB_2928829
APC anti-CD133	Miltenyi Biotec	#130-113-106<br>RRID: AB_2725935
PE-Vio 770 anti-CD133	Miltenyi Biotec	#130-113-110<br>RRID: AB_2725939
FITC anti-CD49a	Miltenyi Biotec	#130-119-305<br>RRID: AB_2733384
GLUCAGON	Cell Signaling	#8233<br>RRID: AB_10859908
NKX6.1	DSHB	#F55A12<br>RRID: AB_532379
**Chemicals, peptides, and recombinant proteins**		
CHIR99021	Selleck	#S1263
Activin A	PeproTech	#120-14
KGF	PeproTech	#100-19
Vitamin C	Sigma Aldrich	#A4403
LDN193189	Tebu Tech	#04-0074-10
SANT1	Sigma Aldrich	#S4572
Retinoic acid	Sigma Aldrich	#R2625
TBP	Calbiochem	#565740
ALK5iII	Santa Cruz	#sc-221234A
γ-secretase inhibitor XX (γSec-iXX)	Merck Millipore	#565789
SB431542	Selleck	#S1067
Wnt-C59	Selleck	#S7037
EGF	PeproTech	#AF-100-15
ISX9	Selleck	#S7914
Triiodothyronine (T3)	Selleck	#S4217
Zinc sulfate	Sigma	#Z0251
N-Acetyl Cysteine	Sigma	#A9165
Trolox	Merck Millipore	#648471
R428	Med Chem Express	#HY-15150
Bovine serum albumin (BSA)	Sigma Aldrich	#B4287

45 % glucose solution in water	Sigma Aldrich	#G8769
7.5% sodium bicarbonate solution	Thermo Fisher	#25080094
GlutaMAX™ Supplement	Thermo Fisher	#35050061
Penicillin-Streptomycin	Thermo Fisher	#15140122
Heparin	Sigma Aldrich	#H3149-100KU
Y-27632	Merck Millipore	#688000
Biolaminin 521 LN (LN521)	BioLamina	#LN521-05
hESC-Qualified Matrigel	Merck	#CLS354277
Growth factor-reduced Matrigel	Merck	#CLS356231
B27 supplement minus Insulin	Thermo Fisher	#A1895601
B27 supplement	Thermo Fisher	#17504001
Exendin-4	Sigma Aldrich	#E7144
**Critical commercial assays**		
RNeasy Micro Kit	Qiagen	#74004
iScript cDNA Synthesis Kit	Bio-Rad	#1708891
LIVE/DEAD™ Fixable Violet Dead Cell Stain Kit	Thermo Fisher	#L34964
Ultrasensitive C-peptide ELISA	Mercodia	#10-1141-01
Insulin ELISA	Mercodia	#10-1113-10
**Experimental models: Cell lines**		
SA121	Takara bio	RRID: CVCL_B296
H9	WiCell	RRID: CVCL_9773
NEUROG3-GFP	Löf-Öhlin, et al (Lof-Ohlin et al., 2017)	NA
MODY3-iPSC-1-CorB	Hermann, et al (Hermann et al., 2023)	NA
**TaqMan™ probes**		
RPL37A	Thermo Fisher	Hs99999909
INS	Thermo Fisher	Hs02741908
GCG	Thermo Fisher	Hs01031536
SST	Thermo Fisher	Hs00356144
TPH1	Thermo Fisher	Hs00188220
SLC18A1	Thermo Fisher	Hs00915193
SOX9	Thermo Fisher	Hs00165814
CFTR	Thermo Fisher	Hs00357011
MAFA	Thermo Fisher	Hs01651425
UCN3	Thermo Fisher	Hs00846499
**Software and algorithms**		
Fiji 2.0/ImageJ	NIH Image	http://imagej.nih.gov/ij
GraphPad Prism 10	GraphPad	https://www.graphpad.com
FlowJo 10	BD Biosciences	https://www.flowjo.com

hESC differentiation

Protocol A: hPSCs were seeded on growth factor-reduced Matrigel-coated T75 flasks at a density of 1.6 × 10^5 cells/cm² for pancreatic differentiation. After 24 hours, differentiation toward pancreatic lineages was initiated following a modified protocol based on Rezania et al.(Hermann et al., 2023). On day 13, cells were dissociated and re-aggregated in 6-well suspension plates at 5 × 10^6 cells per well on an orbital shaker at 100 rpm. On day 20, cells

were sorted using cell surface antibodies, then re-aggregated in AggreWell™ 400 plates (STEMCELL Technologies, #34450) at 1.5 × 10^6 cells per well or Elplasia 96-well round-bottom ultra-low attachment plate (Corning, #4442) at 0.2 × 10^6 cells per well in Stage 6 basal medium. After 24 hours, aggregates were transferred to 6-well suspension plates and cultured for an additional 6 days in Stage 6 basal medium. For in vitro maturation, cells are cultured in Stage 7 medium for 10 days, with medium changed every other day.

Protocol B: hESCs were seeded on LN521-coated T75 flasks at a density of 1.6 × 10^5 cells/cm^2 for pancreatic differentiation. After 24 hours, differentiation toward pancreatic lineages was initiated following a protocol Du et al.(Du et al., 2022) with the following modifications: i) in Stage 1, B27 minus insulin was used in place of standard B27; (ii) on Stage 1, day 1, the medium was devoid of PI103 and Y-27632; (iii) on Stage 1, day 1, the CHIR99021 concentration was reduced from 6 µM to 3 µM; and (iv) on Stage 1, days 2-4, the Activin A concentration was increased from 50 ng/ml to 100 ng/ml. On day 25, cells were sorted using cell surface antibodies, then re-aggregated in AggreWell™ 400 plates (STEMCELL Technologies, #34450) at 1.5 × 10^6 cells per well in Stage 6 basal medium. After 24 hours, aggregates were transferred to 6-well suspension plates and cultured for an additional 6 days in Stage 6 basal medium.

The use of human pluripotent stem cell (hPSC) lines

SA121 (XY, RRID: CVCL_B296), WA09 (H9, XX, RRID: CVCL_9773) and NEUROG3-GFP (XY) hESC lines, and MODY3-iPSC-1-CorB (the corrected MODY3 mutant from the MODY3 patient-derived iPSC line, XX) iPSC line were used in this paper. The SA121 cell line is from Takara Bio (Y00020), the H9 cell line is from WiCell, the NGRUOG3-GFP cell line is derived from SA121 in our previous work(Lof-Ohlin et al., 2017), and the MODY3-iPSC-1-CorB cell line was generated in our previous work(Hermann et al., 2023). The karyotype of hPSCs was normal. For protocol A differentiation: all hPSC lines were cultured on hESC-Qualified Matrigel according to the manufacturer's protocol with daily changes of mTESR™ plus Medium (STEMCELL Technologies, #100-0276) at 37 °C and 5% CO_2. For protocol B differentiation: H9 cell line was cultured on Biolaminin 521 LN (LN521) according to the manufacturer's protocol with daily changes of mTESR™ plus Medium at 37 °C and 5% CO_2. Cells were passaged twice to three times (seeding as 40,000 -60,000 cells/cm^2) weekly at subconfluency using Accutase (Innovative Cell Technologies, #AT-107). The medium was supplemented with 10 µM ROCK inhibitor (Y-27632) on the first day post-passaging. hPSCs were frozen by CryoStor cryopreservation media (Sigma, #C2874). hPSCs were sterile and tested negative for mycoplasma in regular tests. The hPSCs undergo regular checks of the pluripotency by flow cytometry. All experiments were conducted using cells within 2 to 10 passages after thawing.

Flow cytometry analysis

Cells were dissociated by Accutase™. The LIVE/DEAD™ fixable blue dead cell stain kit (Thermo Fisher, #L34964) distinguishes live and dead cells. Then, cells were fixed and stained as previously described(Mamidi et al., 2018) on a Miltenyi MACSQuant analyzer. Antibodies are listed in the Key Resource Table. Data were analyzed using FlowJo software (FlowJo LLC) with established gating strategies. The mean CD133 expression was calculated in FlowJo as the total CD133 signal divided by the number of analyzed cells. Percentile and interquartile range values were calculated using the built-in statistics function in FlowJo.

Immunofluorescence staining

Aggregates were embedded in the hydrogel (Sigma, #TRUE5) on the 8-well chamber (ibidi, # 80827) according to the manufacturer's instructions. Fixation and immunological staining of hESC differentiated cells were performed as previously described(Mamidi *et al.*, 2018). Cells were fixed in 3.7% formalin for 20 minutes, permeabilized with 0.5% Triton X-100 for 60 minutes, and blocked with 5% donkey serum for 2 hours. Primary antibodies are listed in the Key Resource Table. All Alexa Fluor-conjugated secondary antibodies (Thermo Fisher) were used as 1: 500 dilutions. Samples were imaged with Zeiss LSM780 confocal microscopes or the X-light V3 (CrestOptics) spinning disk with the Hamamatsu ORCA-flash camera. Image analyses were performed with Fiji (ImageJ).

RT-qPCR

Total RNA was extracted using the RNeasy Micro Kit. According to the manufacturer's instructions, reverse transcription was performed with iScript cDNA Synthesis Kit. Real-time PCR measurements were performed in technical duplicates using the QuantStudio 7 Flex Real-Time-PCR-System (Thermo Fisher) with TaqMan FAM probes (Thermo Fisher) and TaqMan Master Mix (Thermo Fisher, #4364103). Relative gene expression was determined using the housekeeping genes *RPL37A*. TaqMan probes and primers are listed in the Key Resource Table.

Insulin content and glucose-stimulated insulin secretion (GSIS) assays

On the day of the GSIS assays, 20-40 aggregates were transferred into the low-binding 1.5 ml EP tubes, and then washed twice with Krebs-Ringer bicarbonate buffer (KRB) containing 115 mM NaCl, 5 mM KCl, 1 mM $MgCl_2$, 2.2 mM $CaCl_2$, 20 mM HEPES, 24 mM $NaHCO_3$ and 0.2 % BSA, and pH adjusted to 7.4. All subsequent incubation steps were carried out at 37 °C and 5% CO_2. For GSIS assays, the cells were pre-incubated for 1.5 hrs in KRB supplemented with 1.67 mM glucose before consecutive 30 min incubations in the first KRB with low glucose (2 mM), high glucose (16.67 mM), low glucose (1.67 mM), high glucose plus 10 nM Exendin4 (Ex4) and finally low glucose with KCl (30 mM total). After each step, the medium was

collected, and the cells were washed twice with KRB. Finally, the aggregates were collected, washed once with PBS, and dissociated using Accutase. Half of the cells were used for counting, and the other half were sonicated in H_2O until the solution became clear (4 °C, 20 sec on with 20 sec off). Then, 50 µl of the sonicated solution was transferred into 125 µl 95% HCl-EtOH for insulin content measurement. The remaining sonicated solution was used for genomic DNA quantification. All samples were stored at -80 °C until analysis in technical duplicates with commercially available ELISA kits for Insulin content and secreted human CPEP according to the manufacturer's recommendations.

Reference

Du, Y., Liang, Z., Wang, S., Sun, D., Wang, X., Liew, S.Y., Lu, S., Wu, S., Jiang, Y., Wang, Y., et al. (2022). Human pluripotent stem-cell-derived islets ameliorate diabetes in non-human primates. Nat Med *28*, 272-282. 10.1038/s41591-021-01645-7.

Hermann, F.M., Kjaergaard, M.F., Tian, C., Tiemann, U., Jackson, A., Olsen, L.R., Kraft, M., Carlsson, P.O., Elfving, I.M., Kettunen, J.L.T., et al. (2023). An insulin hypersecretion phenotype precedes pancreatic beta cell failure in MODY3 patient-specific cells. Cell Stem Cell *30*, 38-51 e38. 10.1016/j.stem.2022.12.001.

Lof-Ohlin, Z.M., Nyeng, P., Bechard, M.E., Hess, K., Bankaitis, E., Greiner, T.U., Ameri, J., Wright, C.V., and Semb, H. (2017). EGFR signalling controls cellular fate and pancreatic organogenesis by regulating apicobasal polarity. Nat Cell Biol *19*, 1313-1325. 10.1038/ncb3628.

Mamidi, A., Prawiro, C., Seymour, P.A., de Lichtenberg, K.H., Jackson, A., Serup, P., and Semb, H. (2018). Mechanosignalling via integrins directs fate decisions of pancreatic progenitors. Nature *564*, 114-118. 10.1038/s41586-018-0762-2.
