# Supplementary material for: CD133/CD49a discriminate between human pluripotent stem cell-derived pancreatic beta and alpha cells
Source: Stem Cell Reports. 2026 Mar 5;21(4):102828. doi: 10.1016/j.stemcr.2026.102828 (PMC13083789; doi:10.1016/j.stemcr.2026.102828)
Supplement: Document S2. Article plus supplemental information [file mmc2.pdf]

STEM CELL REPORTS
Report
OPEN ACCESS

CD133/CD49a discriminate between human pluripotent stem cell-derived pancreatic beta and alpha cells

Chenglei Tian,[1,3,*] Yilin Di,[1,2,3] Aisha Muhammad,[1] and Henrik Semb[1,4,*]
[1]Institute of Translational Stem Cell Research, Helmholtz Diabetes Center, Helmholtz Zentrum Munchen, Munich, Germany
[2]School of Medicine and Health, Technical University of Munich, Munich, Germany
[3]These authors contributed equally
[4]Lead contact
*Correspondence: chenglei.tian@helmholtz-munich.de (C.T.), henrik.semb@helmholtz-munich.de (H.S.)
https://doi.org/10.1016/j.stemcr.2026.102828

SUMMARY

Human pluripotent stem cell (hPSC)-derived pancreatic beta cells provide an unlimited cell source for disease modeling and drug development. Generating highly purified beta cell populations from hPSCs remains challenging due to contamination by off-target and polyhormonal cells. Here, we present a robust cell-sorting-based purification strategy to enhance stem cell-derived beta (SC-beta) cell purity. Building on our previous work, we identified CD133 (PROM1) as a beta cell-enriched surface marker capable of distinguishing SC-beta cells from SC-alpha cells. Combining CD133 with the pan-endocrine marker CD49a (ITGA1) significantly increased beta cell enrichment while drastically reducing the fractions of alpha cells, polyhormonal cells, and ductal cells. This effect was consistent across multiple hPSC lines and differentiation protocols. Our approach yields SC-beta cell preparations with markedly improved purity, thereby advancing their application in disease modeling and drug development.

INTRODUCTION

Stem cell (SC)-derived pancreatic beta cells provide a renewable and scalable cell source for physiologically relevant disease models of diabetes, enabling the study of pathogenic mechanisms in a human context (Cujba et al., 2022; Hermann et al., 2023; Li et al., 2024; Singh et al., 2025). Moreover, these cells offer a powerful platform for drug discovery and development, facilitating the screening and evaluation of therapeutic candidates (Hu et al., 2025).

Over the past decade, stepwise differentiation protocols have enabled the generation of SC-beta cells (Balboa et al., 2022; Du et al., 2022; Rezania et al., 2014; Russ et al., 2015; Velazco-Cruz et al., 2019). However, achieving high purity in SC-beta cell preparations remains a significant challenge. The by-product populations, particularly alpha cells and polyhormonal cells (eventually will become alpha cells; Augsornworawat et al., 2023; Hiyoshi et al., 2024; Hiyoshi et al., 2022; Nair et al., 2019), can alter beta cell function through intercellular interactions, thereby limiting the use of SC-beta cells in disease modeling and drug development.

To address these concerns, efforts have been made to enrich beta cells using insulin reporter lines and antibody-based sorting strategies (Docherty et al., 2021; Nair et al., 2019; Parent et al., 2022; Saunders et al., 2019; Veres et al., 2019). Reporter-based approaches are highly effective but depend on genetic modification, which limits their translational potential. Antibody-based methods provide a more clinically relevant alternative; however, many rely on lab-generated reagents that are not commercially accessible and require additional validation (Docherty et al., 2021; Parent et al., 2022; Saunders et al., 2019). CD49a (ITGA1), identified through single-cell RNA sequencing, has recently been proposed as a commercially available marker for beta cell enrichment (Veres et al., 2019). Nevertheless, its specificity remains suboptimal (Molakandov et al., 2021), restricting its utility to broader endocrine enrichment (including alpha cells and polyhormonal cells) rather than selective beta cell purification. In addition, ENTPD3 can be used to enrich SC-beta cells, but it primarily marks a more mature and functional population (Docherty et al., 2021). Moreover, CD99 enriches human adult beta cells (Martens et al., 2018), and CD71 enriches mouse adult beta cells (Berthault et al., 2020), but neither marker has been validated in SC-beta cells.

Recent work has highlighted apical-basal polarity as a critical determinant of islet lineage specification (Tiemann et al., 2025; Tyler, 2003). As EPs mature, beta cells temporarily retain a diminished apical domain, while alpha cells remain non-polarized (Lof-Ohlin et al., 2017; Nyeng et al., 2019). Prominin-1 (PROM1, CD133), a transmembrane glycoprotein localized at apical membranes, has been identified as a marker of polarity, raising the possibility that it could distinguish immature beta cells from alpha and polyhormonal cells (Corbeil et al., 2010; Tiemann et al., 2025).

Here, we present a cell sorting-based strategy that combines CD133 and CD49a antibodies to enrich SC-beta cells. This dual-marker approach achieves more than 70% purity while eliminating off-target populations and alpha cells.

Published by Elsevier Inc. on behalf of International Society for Stem Cell Research.
This is an open access article under the CC BY license (http://creativecommons.org/licenses/by/4.0/).

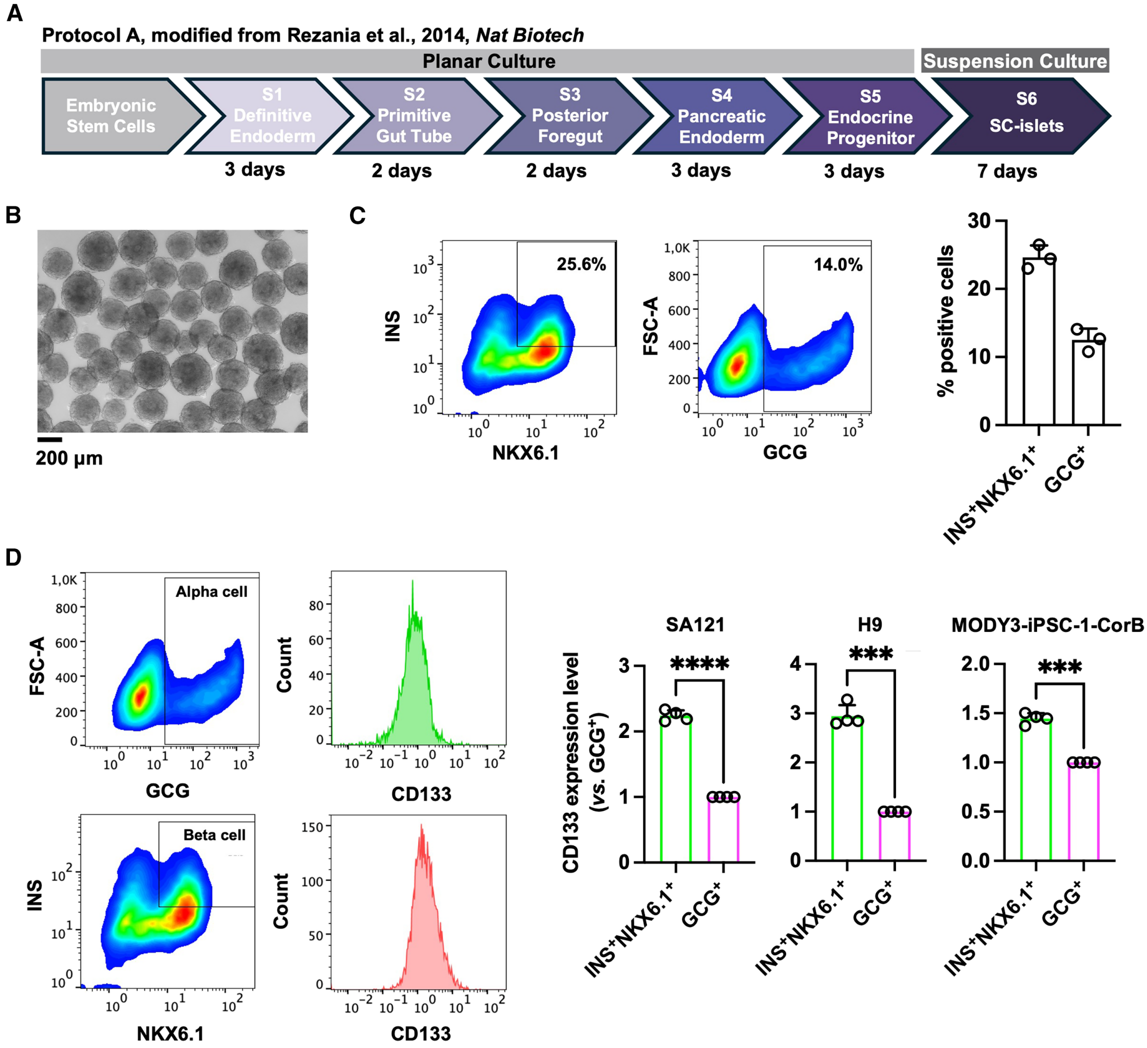

Figure 1. CD133 is a beta cell-enriched surface marker within SC-islets

(A) Schematic diagram of a stepwise differentiation protocol to generate SC-islets from hPSCs. The protocol is modified from Rezania et al. *Nat Biotech* (Rezania et al., 2014).

(B) Representative bright-field morphology images of SC-islets at the end of stage 6. Scale bars, 200 μm. Image is from the SA121 cell line.

(C) Representative flow cytometry plots (left) and the quantification (right) of INSULIN $(INS)^+NKX6.1^+$ beta cells and Glucagon $(GCG)^+$ alpha/polyhormonal cells in SC-islets at the end of differentiation. Data are presented as the mean ± SD ($n = 3$). Data are from SA121 ($n = 1$), H9 ($n = 1$), and MODY3-iPSC-1-CorB ($n = 1$) cell lines.

(D) Representative flow cytometry plots (left) and the quantification (right) of CD133 expression between alpha/polyhormonal and beta cells at the end of differentiation. CD133 expression level is normalized by GCG^+ cells. Data are presented as the mean ± SD ($n = 4$). ***$p < 0.001$; ****$p < 0.0001$. Data are from SA121, H9, and MODY3-iPSC-1-CorB cell lines.

RESULTS

CD133 is a beta cell-enriched cell surface marker in SC-islets

Within SC-islets, alpha cells and beta cells are the predominant endocrine populations. We investigated whether CD133, a polarity-associated surface protein, could distinguish these populations. Using a modified Rezania et al. protocol (Rezania et al., 2014) (protocol A; Figure 1A), three genetically distinct human pluripotent stem cell (hPSC) lines (SA121, H9, and MODY3-iPSC-1-CorB; Figures S1A–S1C) generated comparable alpha/polyhormonal (marked by GCG^+) and beta cell (marked $INS^+NKX6.1^+$) differentiation efficiencies at day 7 of stage 6 (Figures 1B, 1C, and S2). Across all lines, CD133 expression was consistently and significantly higher in beta cells than in alpha/polyhormonal cells, identifying CD133 as a beta cell-enriched marker (Figure 1D).

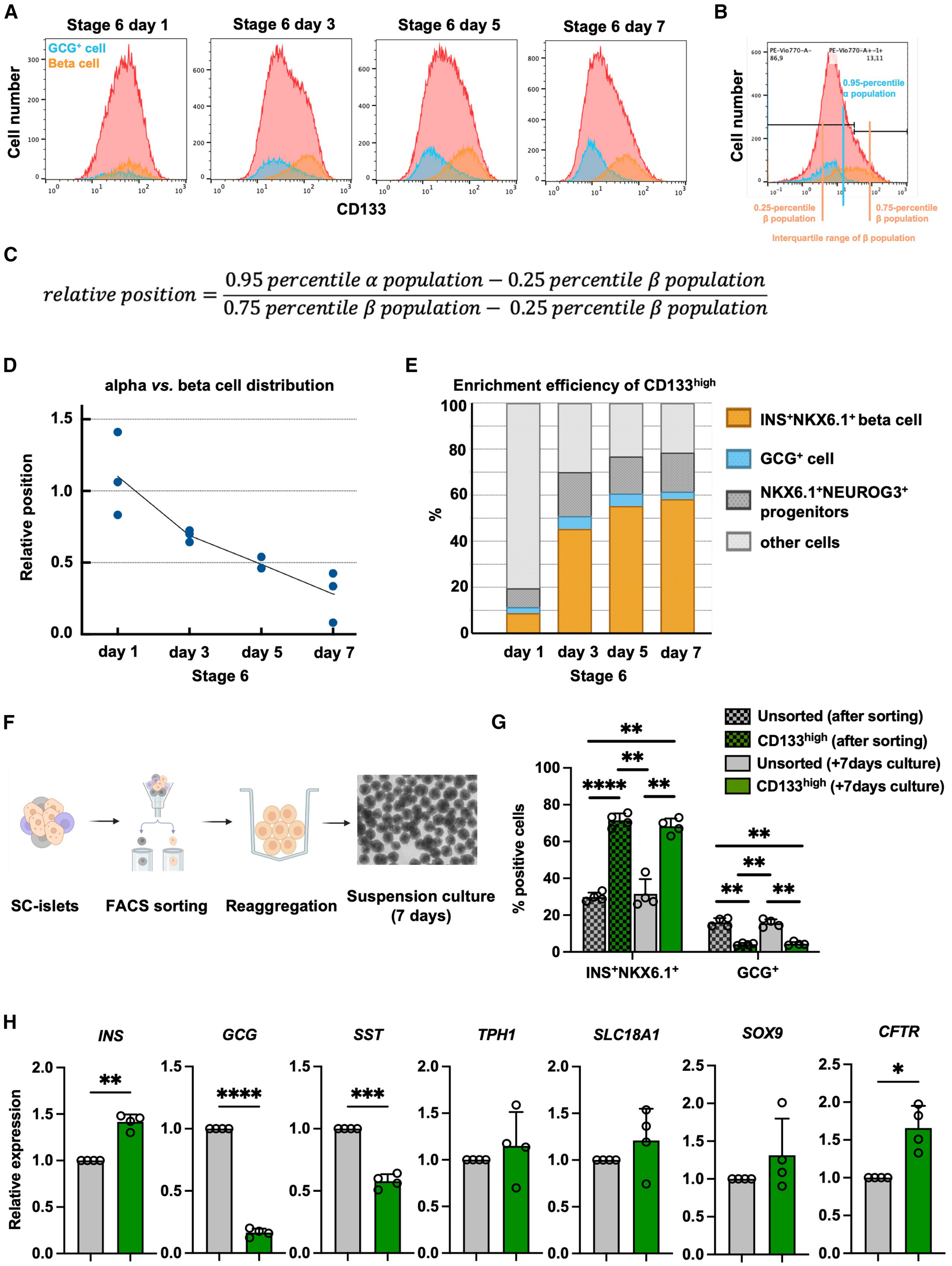

Figure 2. CD133 as a cell surface marker for excluding glucagon-producing cells and enhancing the purity of SC-derived beta cells

(A) Time course of GCG$^+$ alpha/polyhormonal cells (blue) and INS$^+$NKX6.1$^+$ beta cells (orange) distribution among all cell populations (red) during stage 6 differentiation. NEUROG3$^+$ cells, representing the overall endocrine population, were identified as the red histogram. Within this population, GCG$^+$ cells were depicted in blue, and INS$^+$NKX6.1$^+$ cells in orange. All population histograms were plotted according to

(legend continued on next page)

In addition, CD133 expression in beta cells decreased during *in vitro* maturation (Figures S3A and S3B), consistent with reports showing lower CD133 (encoded by *PROM1*) levels in *in vivo*-grafted (mature) versus *in vitro*-derived (immature) SC-beta cells (Augsornworawat et al., 2020), suggesting that CD133 marks newly formed, immature beta cells.

CD133-based sorting enriches beta and ductal-like cells

Flow cytometry analysis integrated alpha/polyhormonal and beta subpopulations according to CD133 expression intensity, enabling clear visualization of their temporal dynamics (Figure 2A). As the EP stage represents a dynamically shifting microenvironment where progenitors continually alter their polarity status (Tiemann et al., 2025), the timing of cell sorting is critical for achieving high enrichment efficiency. To strengthen the analysis, the 0.95 percentile of the alpha/polyhormonal population was used as the threshold to define its upper boundary, reflecting the aim of excluding this population. In contrast, the interquartile range of the beta population was applied to characterize the distribution of beta cells (Figure 2B). Based on the algorithm (Figure 2C), a higher relative position index indicates greater overlap between the two populations, whereas values closer to 0 reflect minimal overlap. During stage 6 differentiation, alpha/polyhormonal and beta cell populations became progressively more distinct (Figure 2D). By day 7 of stage 6, a clear separation was observed, with the NEUROG3^{+}CD133^{high} population representing a substantial fraction of beta cells (Figure 2E). These findings suggest that adjusting sorting to this window could yield optimal enrichment efficiency.

To evaluate the utility of CD133 for beta cell purification, stage 6 day 7 SC-islets were sorted into CD133^{high} and unsorted populations, reaggregated, and maintained in suspension culture for 7 days (Figure 2F). Our results showed that CD133 expression was higher in beta cells than in GCG^{+} alpha/polyhormonal cells (Figure 1D). As our differentiation protocol typically yields 20%–30% beta cells (Figure 1C), we set the CD133^{high} sorting threshold at 20% to efficiently and consistently enrich beta cells (Figure S4A). Flow cytometry revealed that the CD133^{high} sorted cells contained a significantly higher proportion of INS^{+}NKX6.1^{+} beta cells and a markedly reduced proportion of GCG^{+} alpha/polyhormonal cells compared with unsorted controls (Figure 2G). In addition, because most progenitor fates are determined by the end of stage 6, the proportions of INS^{+}NKX6.1^{+} beta cells and GCG^{+} alpha cells remained stable following re-aggregation and 7 days of suspension culture (Figure 2G). Consistent with these findings, quantitative real-time PCR (RT-qPCR) analysis showed elevated INS expression and reduced GCG and SST transcripts in CD133^{high} cells (Figure 2H), indicating effective enrichment of beta cells and depletion of other types of endocrine cells.

However, CD133^{high} sorting also led to enrichment of CFTR-expressing cells, a marker of ductal-like cells, as well as modest increases in SOX9, another ductal progenitor marker (Figure 2H). This is consistent with the known fact that ductal cells, like newborn beta cells, are polarized. These results suggest that while CD133 is effective for beta cell enrichment and the removal of alpha and polyhormonal cells, additional markers are required to exclude ductal contaminants from the purified population.

Combining CD133 with CD49a eliminates ductal contaminants, thereby increasing beta cell purity

CD49a (ITGA1) is a pan-endocrine cell surface marker that is broadly expressed in endocrine cells but absent from ductal epithelium (Veres et al., 2019), making it a suitable candidate for excluding CD133^{+} ductal contaminants.

CD133 intensity. Overlaying these subpopulations on the total endocrine population enabled visualization of lineage segregation based on CD133 expression, with reduced overlap indicating clearer distinction between alpha and beta lineages. Data are from the NEUROG3-GFP cell line.

(B) Illustration of the interquartile range of beta cells and the 0.95 percentile of alpha/polyhormonal cells.

(C) Algorithm to define the relative position index. Values approaching 1 indicated substantial overlap, while values approaching 0 reflected distinct separation.

(D) Alpha cell vs. beta cell relative position index based on CD133 expression level along stage 6 differentiation. Data are from the NEUROG3-GFP cell line.

(E) Flow cytometry analysis of GCG^{+} alpha/polyhormonal cells, INS^{+}NKX6.1^{+} beta cells, NKX6.1^{+}NEUROG3^{+} progenitors, and other cells based on gating selection during stage 6 differentiation. Data are from the NEUROG3-GFP cell line.

(F) Schematic diagram of the cell sorting, reaggregation, and subsequent suspension culture. At the end of differentiation, cells were sorted based on CD133 expression. The sorted cells were then reaggregated, and one day after reaggregation, they were transferred to a 6-well suspension culture plate for 7 days of culture. The cells were collected after sorting and after 7 days of culture for analysis.

(G) Flow cytometry analysis of INS^{+}NKX6.1^{+} beta cells and GCG^{+} alpha/polyhormonal cells in unsorted and CD133-sorted populations. Data are presented as the mean ± SD ($n = 4$). **$p < 0.01$; ****$p < 0.0001$. Data are from the SA121 cell line.

(H) RT-qPCR assessment of marker genes in unsorted and CD133-sorted populations. Data are presented as the mean ± SD ($n = 4$). *$p < 0.05$; **$p < 0.01$; ***$p < 0.001$; ****$p < 0.0001$. Data are from the SA121 cell line.

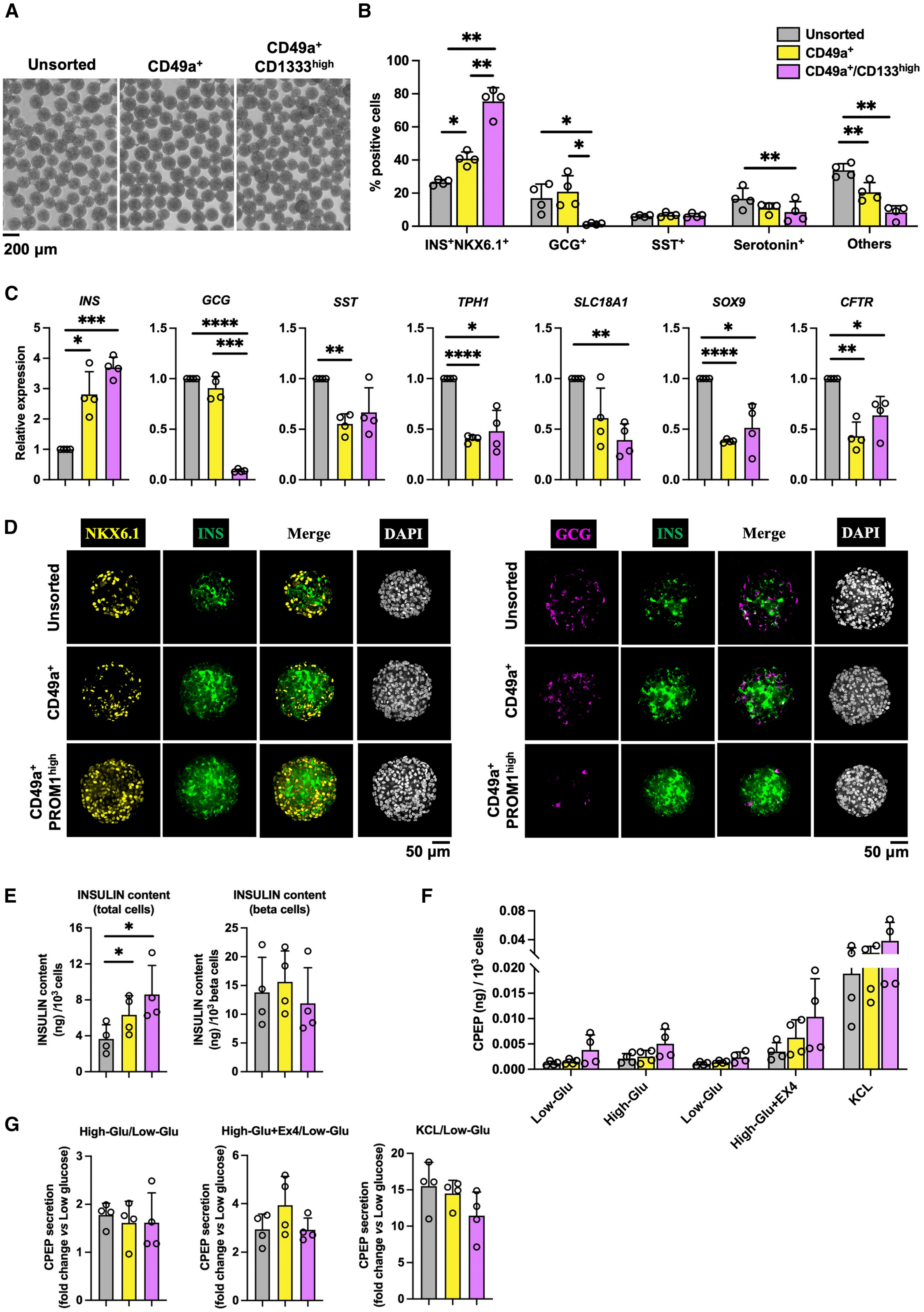

(legend on next page)

Because the endocrine cell composition remained stable for 7 days after re-aggregation (Figure 2B), only cells at this time point were analyzed. Compared with unsorted and CD49a alone-sorted cells, dual CD49a/CD133 selection yielded the highest beta cell purity (>75% INS$^+$NKX6.1$^+$) and most efficient removal of GCG$^+$ alpha and polyhormonal cells (Figures 3B and 3C). In addition, CD49a/CD133-sorted cells show a significant reduction of enterochromaffin-like cells compared with unsorted cells (Figures 3B and 3C). Importantly, ductal markers *CFTR* and *SOX9* were markedly reduced in the CD49a alone and dual CD49a/CD133-sorted population, confirming effective removal of ductal-like contaminants (Figure 3C). Immunofluorescence analysis corroborated these results, showing a predominance of INS$^+$NKX6.1$^+$ beta cells and near-complete absence of GCG$^+$ alpha and polyhormonal cells in the dual CD49a/CD133-sorted population (Figure 3D).

Additionally, the higher beta cell proportion in dual CD49a/CD133-sorted populations resulted in an overall higher total insulin content compared with unsorted or CD49a-only sorted cells, but did not increase the insulin content per beta cell (Figure 3E). These cells secreted C-peptide in response to high glucose and exendin-4 stimulation (Figure 3F). However, the glucose stimulation index did not differ significantly among unsorted, CD49a-only, and dual-sorted groups, indicating that CD49a and/or CD133 selection enriches beta cell number but does not distinguish between functionally mature and less mature beta cells.

Dual CD49a/CD133 sorting reproducibly enriches beta cells across hPSC lines and differentiation protocols

To assess the robustness of the dual-marker sorting strategy, we used the H9 hESC and MODY3-iPSC-1-CorB lines differentiated under protocol A (Rezania et al., 2014) (Figures S5 and S6). In each setting, dual CD49a/CD133 sorting consistently enriched beta cells to >70% purity, minimized alpha/polyhormonal, enterochromaffin-like, and ductal-like cells, and improved total insulin content (Figures S5A–S5D and S6A–S6D). The glucose stimulation index remained comparable across groups, consistent with results from the SA121 line (Figures S5E and S6E).

The reproducibility of this approach was further tested by applying CD49a/CD133 sorting to SC-islets generated using an alternative differentiation protocol (protocol B, modified from Du et al., 2022; Figures S7A and S7B). As observed with protocol A, dual CD49a/CD133 sorting markedly increased the proportion of INS$^+$NKX6.1$^+$ beta cells while reducing alpha/polyhormonal, enterochromaffin-like, and ductal-like cell contaminants (Figure S7C). Transcript analysis confirmed elevated *INS* expression with reduced *GCG*, *TPH1*, *SLC18A1*, *SOX9*, and *CFTR* levels in dual-sorted populations (Figure S7D), accompanied by increased insulin content (Figure S7E) and proper GSIS performance (Figure S7F). In addition, testing CD133 antibody dilutions from 1:5 to 1:20 showed comparable beta cell sorting efficiencies (Figure S8).

Together, these findings demonstrate that CD49a/CD133 dual-marker selection is a robust and generalizable strategy for beta cell enrichment, effective across multiple hPSC lines, and differentiation protocols.

DISCUSSION

In this study, we establish a new cell sorting strategy for the purification of SC-beta cells. Compared with existing methods, our approach provides three main

Figure 3. Enhancing the purity of SC-derived beta cells through CD133 and CD49a-based sorting

(A) Representative bright-field images of unsorted, CD49a-sorted, and CD49a/CD133-sorted cells after 7 days of reaggregation. Scale bars, 200 μm.

(B) Flow cytometry analysis of INS$^+$NKX6.1$^+$ beta cells, GCG$^+$ alpha/polyhormonal cells, Somatostatin (SST)$^+$ delta cells, Serotonin$^+$ enterochromaffin-like cells, and other cells in unsorted, CD49a-sorted and CD49a/CD133-sorted populations. Staining panels included INS + NKX6.1, GCG + SST, and Serotonin. "Other cells" represent all cells excluding INS$^+$NKX6.1$^+$, GCG$^+$, SST$^+$, and Serotonin$^+$ populations. The GCG$^+$ population consists of GCG single-positive alpha cells and GCG$^+$INS$^+$ polyhormonal cells (lack NKX6.1 expression (Peterson et al., 2020; Veres et al., 2019). Data are presented as the mean ± SD ($n = 4$). *$p < 0.05$; **$p < 0.01$.

(C) RT-qPCR assessment of marker genes in unsorted, CD49a sorted, and CD49a/CD133 sorted populations. Data are presented as the mean ± SD ($n = 4$). *$p < 0.05$; **$p < 0.01$; ***$p < 0.001$; ****$p < 0.0001$.

(D) Immunostaining on INS, NKX6.1, and GCG expression in unsorted, CD49a-sorted, and CD49a/CD133-sorted populations. Scale bars, 50 μm.

(E) Insulin content measured by ELISA in unsorted, CD49a-sorted, and CD49a/CD133-sorted cell populations. Left: insulin content normalized by 10^3 total cells; right: insulin content normalized by 10^3 beta cells (total cells/the percentage of beta cells). Data are presented as the mean ± SD ($n = 4$). *$p < 0.05$; **$p < 0.01$.

(F) The C-peptide secretion level after low glucose (Low-Glu), high glucose (Hi-Glu), Low-Glu, high glucose plus Exendin-4 (Hi-Glu+Ex4), and low glucose plus KCl (KCl) treatment. Data are presented as mean ± SD ($n = 4$).

(G) C-peptide secretion stimulation index in static GSIS measured by ELISA in unsorted, CD49a-sorted, and CD49a/CD133-sorted cell populations. Data are presented as the mean ± SD ($n = 4$).

All the data are from the SA121 cell line.

advantages: (1) more effective enrichment of SC-beta cells from the endocrine pool with efficient exclusion of GCG^+ alpha/polyhormonal cells; (2) reliance on cell surface markers compatible with clinical manufacturing workflows, supporting good manufacturing practice (GMP)-compliant implementation; and (3) use of commercially available, well-validated antibodies, thereby enhancing reproducibility and translational feasibility. Together, these advances markedly improve SC-beta cell purity and strengthen their potential for safe and effective application in disease modeling and drug development.

Previous studies have highlighted a critical role for apical-basal polarity in endocrine lineage specification (Tiemann et al., 2025). During mouse pancreatic development, alpha cells are predominantly generated at early stages when the epithelium remains largely non-polarized, whereas beta cells emerge preferentially at later stages, coinciding with the establishment of epithelial polarity and lumen formation (Johansson et al., 2007; Nyeng et al., 2019; Tiemann et al., 2025). Furthermore, live-cell imaging studies revealed that newly formed beta cells transiently retain apical polarity within the epithelium and after delamination (Nyeng et al., 2019). This provided the rationale for employing the apically expressed CD133, an epithelial progenitor marker that is not expressed in mature hormone-positive endocrine cells (Sugiyama et al., 2007), as a surface marker to isolate SC-beta cells.

Still, CD133 alone is not sufficient for the purification of SC-beta cells from a heterogeneous cell population, because it will also target other polarized cells, such as ductal cells (Figures 2G and 2H). To remove ductal cells, we decided to also use the pan-endocrine marker: CD49a, since it is absent from ductal cells (Gharibi et al., 2017; Veres et al., 2019). Based on these properties, we combined CD133 and CD49a to enrich for beta cells while minimizing contamination from ductal cells. To the best of our knowledge, CD133 is the only cell surface marker shown to efficiently enrich immature beta cells, such as SC-beta cells, from the endocrine populations. The fact that CD133, a polarity-associated marker, can distinguish SC-beta cells from alpha/polyhormonal cells further suggests that additional polarity-linked surface markers may be exploited for SC-beta cell purification.

While this study demonstrates that the CD133/CD49a double-sorting strategy provides a robust approach for enriching SC-beta cells, several limitations should be acknowledged. First, this strategy does not achieve complete beta-cell purity, with residual non-beta endocrine cells and progenitor populations remaining, which may raise safety considerations in therapeutic settings and warrant further evaluation in animal transplantation models. Second, it remains to be determined whether the performance of this purification strategy will differ when applied to more efficient beta cell differentiation platforms.

METHODS

Flow cytometry sorting and analysis

Cells were dissociated by Accutase for 20 min at 37°C (10 mL Accutase per 6-well suspension plate). The dissociation was terminated by adding an equal volume of stage 6 basal medium, and the cell suspension was passed through a 40 μm strainer to obtain single cells. The cells were then counted, centrifuged, and resuspended in MACSQuant Tyto Running Buffer (Miltenyi Biotec, #130-107-207) supplemented with CD133 antibody (1:10) and CD49a antibody (1:50) (100 μL buffer per 1×10^6 cells), and incubated for 15 min at room temperature. Subsequently, the cells were washed once with the Running Buffer and sorted on a Miltenyi MACSQuant Tyto sorter using gates to isolate single cells based on scatter characteristics. For $CD133^{high}$ sorting, the gate was set to include the top 20% of high-positive cells; for CD49a, gating was based on the clear separation from the negative population.

Quantification and statistical analysis

Statistical analyses were performed with GraphPad Prism 10 (GraphPad Software). Unless otherwise noted, a paired nonparametric test (Wilcoxon matched-pairs signed-rank test) was used to assess significance. An unpaired nonparametric test was used for unpaired data (Mann-Whitney test). Asterisks denote p values as follows: *$p < 0.05$; **$p < 0.01$; ***$p < 0.001$; ****$p < 0.0001$. Unless otherwise noted, each N represents a biological replicate (one independent differentiation experiment). Data figures illustrate the mean with standard deviation (SD) and the values of individual biological replicates.

RESOURCE AVAILABILITY

Lead contact

Further information and requests for resources and reagents should be directed to and will be fulfilled by the lead contact, Henrik Semb (henrik.semb@helmholtz-munich.de).

Materials availability

All unique/stable reagents in this study are available from the lead contact with a completed Materials Transfer Agreement.

Data and code availability

This study did not generate any custom code. All analyses were performed using standard commercial and open-source software, as detailed in the methods section. All the data collected in this study are available from the lead contact upon a reasonable request.

ACKNOWLEDGMENTS

All the work is supported by the European Union's Horizon 2020 research and innovation program (ISLET, no. 874839); the Federal Ministry of Education and Research (BMBF) project (eISLET, no. 031L0251); and the Helmholtz Zentrum München.

AUTHOR CONTRIBUTIONS

C.T., Y.D., and H.S. designed the experiments; C.T., Y.D., and A.M. performed the experiments; C.T. and Y.D. performed analyses; C.T. and H.S. wrote the manuscript.

DECLARATION OF INTERESTS

C.T. and H.S. are listed as inventors on an international patent application partially based on this work.

SUPPLEMENTAL INFORMATION

Supplemental information can be found online at https://doi.org/10.1016/j.stemcr.2026.102828.

Received: September 12, 2025
Revised: February 2, 2026
Accepted: February 2, 2026
Published: March 5, 2026

REFERENCES

Augsornworawat, P., Hogrebe, N.J., Ishahak, M., Schmidt, M.D., Marquez, E., Maestas, M.M., Veronese-Paniagua, D.A., Gale, S.E., Miller, J.R., Velazco-Cruz, L., and Millman, J.R. (2023). Single-nucleus multi-omics of human stem cell-derived islets identifies deficiencies in lineage specification. Nat. Cell Biol. *25*, 904–916. https://doi.org/10.1038/s41556-023-01150-8.

Augsornworawat, P., Maxwell, K.G., Velazco-Cruz, L., and Millman, J.R. (2020). Single-Cell Transcriptome Profiling Reveals beta Cell Maturation in Stem Cell-Derived Islets after Transplantation. Cell Rep. *32*, 108067. https://doi.org/10.1016/j.celrep.2020.108067.

Balboa, D., Barsby, T., Lithovius, V., Saarimäki-Vire, J., Omar-Hmeadi, M., Dyachok, O., Montaser, H., Lund, P.E., Yang, M., Ibrahim, H., et al. (2022). Functional, metabolic and transcriptional maturation of human pancreatic islets derived from stem cells. Nat. Biotechnol. *40*, 1042–1055. https://doi.org/10.1038/s41587-022-01219-z.

Berthault, C., Staels, W., and Scharfmann, R. (2020). Purification of pancreatic endocrine subsets reveals increased iron metabolism in beta cells. Mol. Metabol. *42*, 101060. https://doi.org/10.1016/j.molmet.2020.101060.

Corbeil, D., Marzesco, A.M., Wilsch-Bräuninger, M., and Huttner, W.B. (2010). The intriguing links between prominin-1 (CD133), cholesterol-based membrane microdomains, remodeling of apical plasma membrane protrusions, extracellular membrane particles, and (neuro)epithelial cell differentiation. FEBS Lett. *584*, 1659–1664. https://doi.org/10.1016/j.febslet.2010.01.050.

Cujba, A.M., Alvarez-Fallas, M.E., Pedraza-Arevalo, S., Laddach, A., Shepherd, M.H., Hattersley, A.T., Watt, F.M., and Sancho, R. (2022). An HNF1alpha truncation associated with maturity-onset diabetes of the young impairs pancreatic progenitor differentiation by antagonizing HNF1beta function. Cell Rep. *38*, 110425. https://doi.org/10.1016/j.celrep.2022.110425.

Docherty, F.M., Riemondy, K.A., Castro-Gutierrez, R., Dwulet, J.M., Shilleh, A.H., Hansen, M.S., Williams, S.P.M., Armitage, L.H., Santostefano, K.E., Wallet, M.A., et al. (2021). ENTPD3 Marks Mature Stem Cell-Derived beta-Cells Formed by Self-Aggregation In Vitro. Diabetes *70*, 2554–2567. https://doi.org/10.2337/db20-0873.

Du, Y., Liang, Z., Wang, S., Sun, D., Wang, X., Liew, S.Y., Lu, S., Wu, S., Jiang, Y., Wang, Y., et al. (2022). Human pluripotent stem-cell-derived islets ameliorate diabetes in non-human primates. Nat. Med. *28*, 272–282. https://doi.org/10.1038/s41591-021-01645-7.

Gharibi, A., La Kim, S., Molnar, J., Brambilla, D., Adamian, Y., Hoover, M., Hong, J., Lin, J., Wolfenden, L., and Kelber, J.A. (2017). ITGA1 is a pre-malignant biomarker that promotes therapy resistance and metastatic potential in pancreatic cancer. Sci. Rep. *7*, 10060. https://doi.org/10.1038/s41598-017-09946-z.

Hermann, F.M., Kjaergaard, M.F., Tian, C., Tiemann, U., Jackson, A., Olsen, L.R., Kraft, M., Carlsson, P.O., Elfving, I.M., Kettunen, J.L.T., et al. (2023). An insulin hypersecretion phenotype precedes pancreatic beta cell failure in MODY3 patient-specific cells. Cell Stem Cell *30*, 38–51.e38. https://doi.org/10.1016/j.stem.2022.12.001.

Hiyoshi, H., Sakuma, K., Asano, S., Napier, S.C., Konagaya, S., Mochida, T., Ueno, H., Watanabe, T., Kassai, Y., Matsumoto, H., et al. (2024). Identification and removal of unexpected proliferative off-target cells emerging after iPSC-derived pancreatic islet cell implantation. Proc. Natl. Acad. Sci. USA *121*, e2320883121. https://doi.org/10.1073/pnas.2320883121.

Hiyoshi, H., Sakuma, K., Tsubooka-Yamazoe, N., Asano, S., Mochida, T., Yamaura, J., Konagaya, S., Fujii, R., Matsumoto, H., Ito, R., and Toyoda, T. (2022). Characterization and reduction of non-endocrine cells accompanying islet-like endocrine cells differentiated from human iPSC. Sci. Rep. *12*, 4740. https://doi.org/10.1038/s41598-022-08753-5.

Hu, R., Ma, Q., Kong, Y., Wang, Z., Xu, M., Chen, X., Su, Y., Xiao, T., He, Q., Wang, X., et al. (2025). A Compound Screen Based on Isogenic hESC-Derived beta Cell Reveals an Inhibitor Targeting ZnT8-Mediated Zinc Transportation to Protect Pancreatic beta Cell from Stress-Induced Cell Death. Adv. Sci. *12*, e2413161. https://doi.org/10.1002/advs.202413161.

Johansson, K.A., Dursun, U., Jordan, N., Gu, G., Beermann, F., Gradwohl, G., and Grapin-Botton, A. (2007). Temporal control of neurogenin3 activity in pancreas progenitors reveals competence windows for the generation of different endocrine cell types. Dev. Cell *12*, 457–465. https://doi.org/10.1016/j.devcel.2007.02.010.

Li, Y., Zheng, R., Jiang, L., Yan, C., Liu, R., Chen, L., Jin, W., Luo, Y., Zhang, X., Tang, J., et al. (2024). A noncoding variant confers pancreatic differentiation defect and contributes to diabetes susceptibility by recruiting RXRA. Nat. Commun. *15*, 9771. https://doi.org/10.1038/s41467-024-54151-y.

Lof-Ohlin, Z.M., Nyeng, P., Bechard, M.E., Hess, K., Bankaitis, E., Greiner, T.U., Ameri, J., Wright, C.V., and Semb, H. (2017). EGFR

signalling controls cellular fate and pancreatic organogenesis by regulating apicobasal polarity. Nat. Cell Biol. *19*, 1313–1325. https://doi.org/10.1038/ncb3628.

Martens, G.A., De Punt, V., and Stangé, G. (2018). CD99 as surface anchor for human islet endocrine cell purification. J. Tissue Eng. Regen. Med. *12*, e171–e176. https://doi.org/10.1002/term.2329.

Molakandov, K., Berti, D.A., Beck, A., Elhanani, O., Walker, M.D., Soen, Y., Yavriyants, K., Zimerman, M., Volman, E., Toledo, I., et al. (2021). Selection for CD26(-) and CD49A(+) Cells From Pluripotent Stem Cells-Derived Islet-Like Clusters Improves Therapeutic Activity in Diabetic Mice. Front. Endocrinol. *12*, 635405. https://doi.org/10.3389/fendo.2021.635405.

Nair, G.G., Liu, J.S., Russ, H.A., Tran, S., Saxton, M.S., Chen, R., Juang, C., Li, M.L., Nguyen, V.Q., Giacometti, S., et al. (2019). Recapitulating endocrine cell clustering in culture promotes maturation of human stem-cell-derived beta cells. Nat. Cell Biol. *21*, 263–274. https://doi.org/10.1038/s41556-018-0271-4.

Nyeng, P., Heilmann, S., Lof-Ohlin, Z.M., Pettersson, N.F., Hermann, F.M., Reynolds, A.B., and Semb, H. (2019). p120ctn-Mediated Organ Patterning Precedes and Determines Pancreatic Progenitor Fate. Dev. Cell *49*, 31–47.e39. https://doi.org/10.1016/j.devcel.2019.02.005.

Parent, A.V., Ashe, S., Nair, G.G., Li, M.L., Chavez, J., Liu, J.S., Zhong, Y., Streeter, P.R., and Hebrok, M. (2022). Development of a scalable method to isolate subsets of stem cell-derived pancreatic islet cells. Stem Cell Rep. *17*, 979–992. https://doi.org/10.1016/j.stemcr.2022.02.001.

Peterson, Q.P., Veres, A., Chen, L., Slama, M.Q., Kenty, J.H.R., Hassoun, S., Brown, M.R., Dou, H., Duffy, C.D., Zhou, Q., et al. (2020). A method for the generation of human stem cell-derived alpha cells. Nat. Commun. *11*, 2241. https://doi.org/10.1038/s41467-020-16049-3.

Rezania, A., Bruin, J.E., Arora, P., Rubin, A., Batushansky, I., Asadi, A., O'Dwyer, S., Quiskamp, N., Mojibian, M., Albrecht, T., et al. (2014). Reversal of diabetes with insulin-producing cells derived *in vitro* from human pluripotent stem cells. Nat. Biotechnol. *32*, 1121–1133. https://doi.org/10.1038/nbt.3033.

Russ, H.A., Parent, A.V., Ringler, J.J., Hennings, T.G., Nair, G.G., Shveygert, M., Guo, T., Puri, S., Haataja, L., Cirulli, V., et al. (2015). Controlled induction of human pancreatic progenitors produces functional beta-like cells *in vitro*. EMBO J. *34*, 1759–1772. https://doi.org/10.15252/embj.201591058.

Saunders, D.C., Brissova, M., Phillips, N., Shrestha, S., Walker, J.T., Aramandla, R., Poffenberger, G., Flaherty, D.K., Weller, K.P., Pelletier, J., et al. (2019). Ectonucleoside Triphosphate Diphosphohydrolase-3 Antibody Targets Adult Human Pancreatic beta Cells for In Vitro and In Vivo Analysis. Cell Metab. *29*, 745–754.e4. https://doi.org/10.1016/j.cmet.2018.10.007.

Singh, T., Kalamajski, S., Cunha, J.P.M.C.M., Hladkou, S., Roberts, F., Gheibi, S., Soltanian, A., Yektay Farahmand, K., Ekström, O., Mamidi, A., et al. (2025). Modeling Genetic Risk of beta-Cell Dysfunction in Human Induced Pluripotent Stem Cells From Patients Carrying the MTNR1B Risk Variant. J. Pineal Res. *77*, e70073. https://doi.org/10.1111/jpi.70073.

Sugiyama, T., Rodriguez, R.T., McLean, G.W., and Kim, S.K. (2007). Conserved markers of fetal pancreatic epithelium permit prospective isolation of islet progenitor cells by FACS. Proc. Natl. Acad. Sci. USA *104*, 175–180. https://doi.org/10.1073/pnas.0609490104.

Tiemann, U., Tian, C., Hermann, F., Proks, M., Skovgaard, E., Kulik, I., Di, Y., Sedzinski, J., and Semb, H. (2025). Pancreatic alpha and beta cell fate choice is directed by apical-basal polarity dynamics. Dev. Cell *60*, 1871–1883.e5. https://doi.org/10.1016/j.devcel.2025.02.008.

Tyler, S. (2003). Epithelium–the primary building block for metazoan complexity. Integr. Comp. Biol. *43*, 55–63. https://doi.org/10.1093/icb/43.1.55.

Velazco-Cruz, L., Song, J., Maxwell, K.G., Goedegebuure, M.M., Augsornworawat, P., Hogrebe, N.J., and Millman, J.R. (2019). Acquisition of Dynamic Function in Human Stem Cell-Derived beta Cells. Stem Cell Rep. *12*, 351–365. https://doi.org/10.1016/j.stemcr.2018.12.012.

Veres, A., Faust, A.L., Bushnell, H.L., Engquist, E.N., Kenty, J.H.R., Harb, G., Poh, Y.C., Sintov, E., Gürtler, M., Pagliuca, F.W., et al. (2019). Charting cellular identity during human *in vitro* beta-cell differentiation. Nature *569*, 368–373. https://doi.org/10.1038/s41586-019-1168-5.

Stem Cell Reports, Volume *21*

Supplemental Information

CD133/CD49a discriminate between human pluripotent stem cell-derived pancreatic beta and alpha cells

Chenglei Tian, Yilin Di, Aisha Muhammad, and Henrik Semb

Supplementary Information

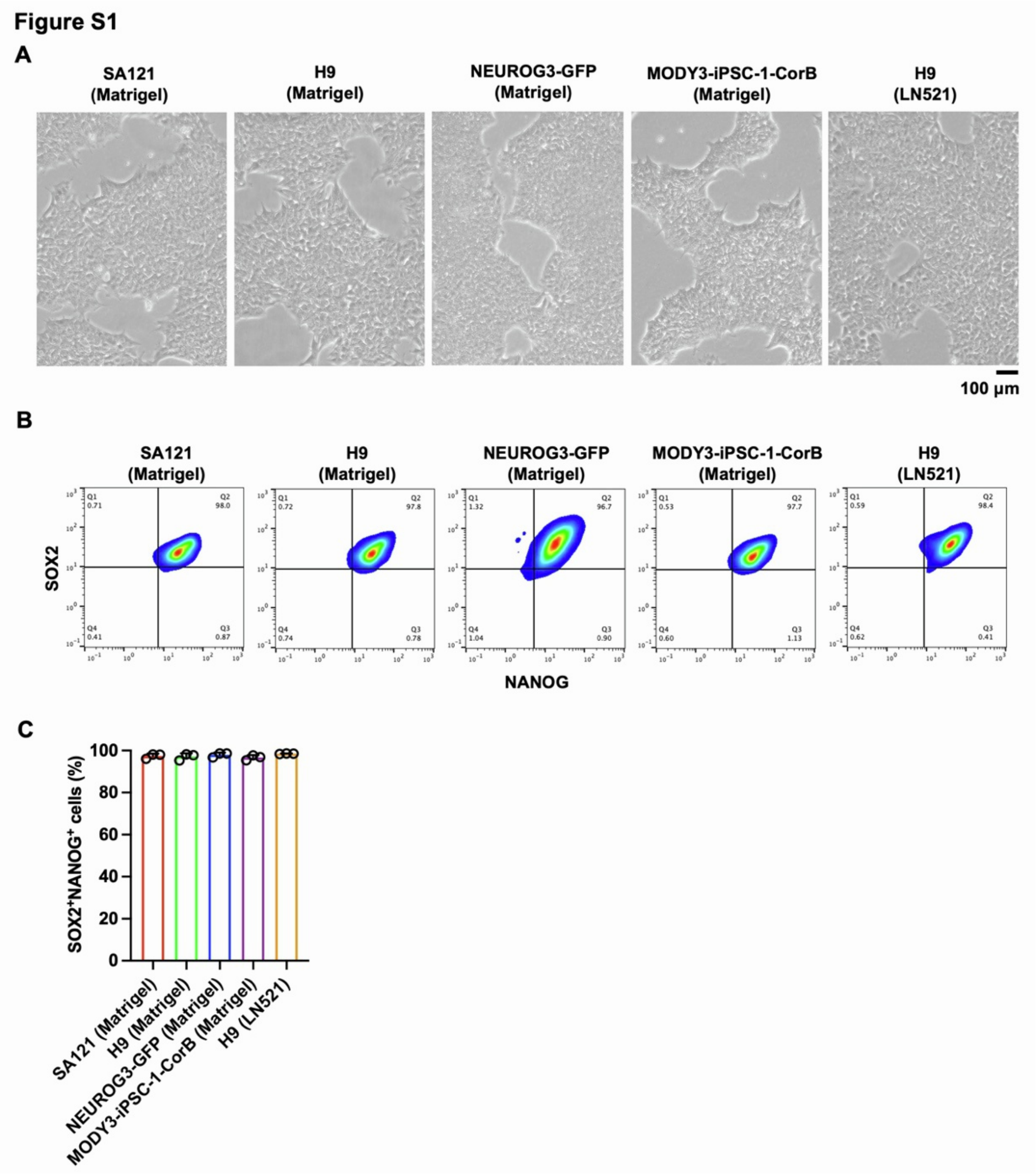

Figure S1. Culture and pluripotency characterization of human pluripotent stem cells (hPSCs), related to Figures 1-3.

(A) Representative bright-field images of Matrigel cultured SA121 human embryonic stem cells (hESCs), H9 hESCs, NEUROG3-GFP hESCs, MODY3-iPSC-1-CorB human induced pluripotent stem cells (hiPSCs), and Laminin 521 (LN521) cultured H9 hESCs. Scale bar, 100 μm.

(B) Representative flow cytometry plots of pluripotent markers (SOX2 and NANOG) in the hPSCs.

(C) The quantification of pluripotent markers (SOX2 and NANOG) in the hPSCs. Data are presented as the mean ± SD (n = 3).

Data are from SA121, H9, NEUROG3-GFP and MODY3-iPSC-1-CorB cell lines.

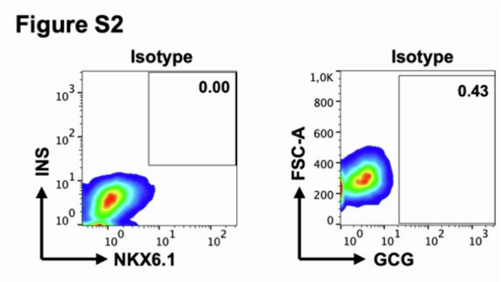

Figure S2. Representative flow cytometry plots of the isotype control for alpha and beta cell gating, related to Figure 1.

Representative flow cytometry plots of the isotype control for Figure 1C. Data are from the SA121 cell line.

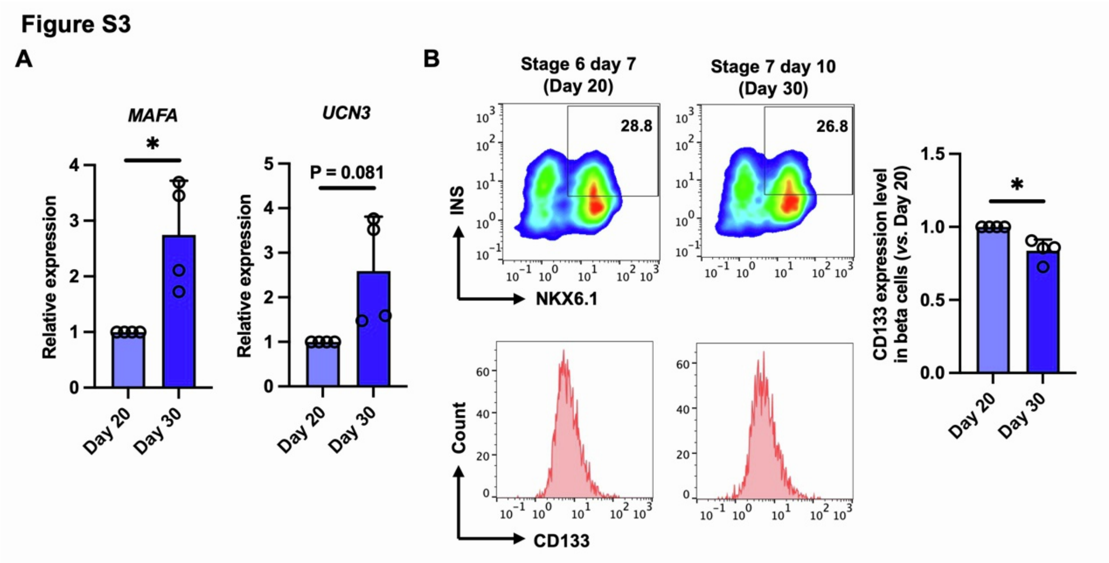

Figure S3. CD133 expression in SC-beta cells during *in vitro* maturation, related to Figure 1.

(A) RT-qPCR assessment of maturation-related genes (*MAFA* and *UCN3*) in Day 20 and Day 30 SC-islets. Data are presented as the mean ± SD (n = 4). *$p < 0.05$.

(B) Representative flow cytometry plots (left) and the quantification (right) of CD133 expression in beta cells before and after *in vitro* maturation. CD133 levels are normalized to Day 20 beta cells. Data are from the SA121 cell line. Data are presented as the mean ± SD (n = 4). *$p < 0.05$.

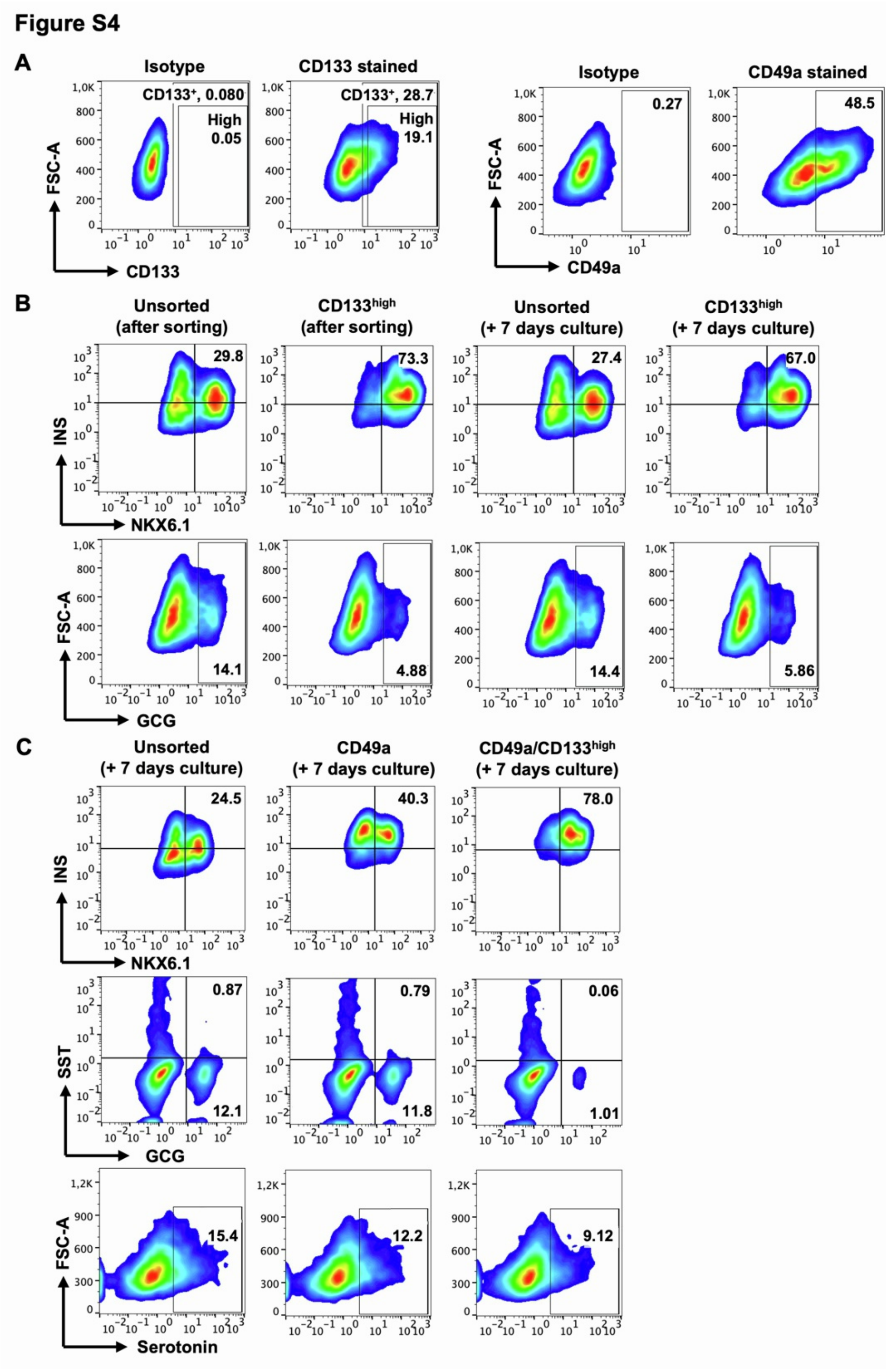

Figure S4. Flow cytometry analysis of purified SC-beta cells, related to Figures 2 and 3.

(A) Representative flow cytometry plots of isotype control, CD133 and CD49a staining of Day 20 samples.

(B) Representative flow cytometry plots of $INS^{+}NKX6.1^{+}$ beta cells and GCG^{+} alpha/polyhormonal cells in unsorted and $CD133^{high}$ populations after sorting and after 7 days of culture.

(C) Representative flow cytometry plots of INS$^+$NKX6.1$^+$ beta cells, GCG$^+$ alpha/polyhormonal cells, SST$^+$ delta cells and Serotonin$^+$ enterochromaffin cells in unsorted, CD49a$^+$ and CD49a$^+$/CD133^{high} populations after 7 days of culture.

Data are from the SA121 cell line.

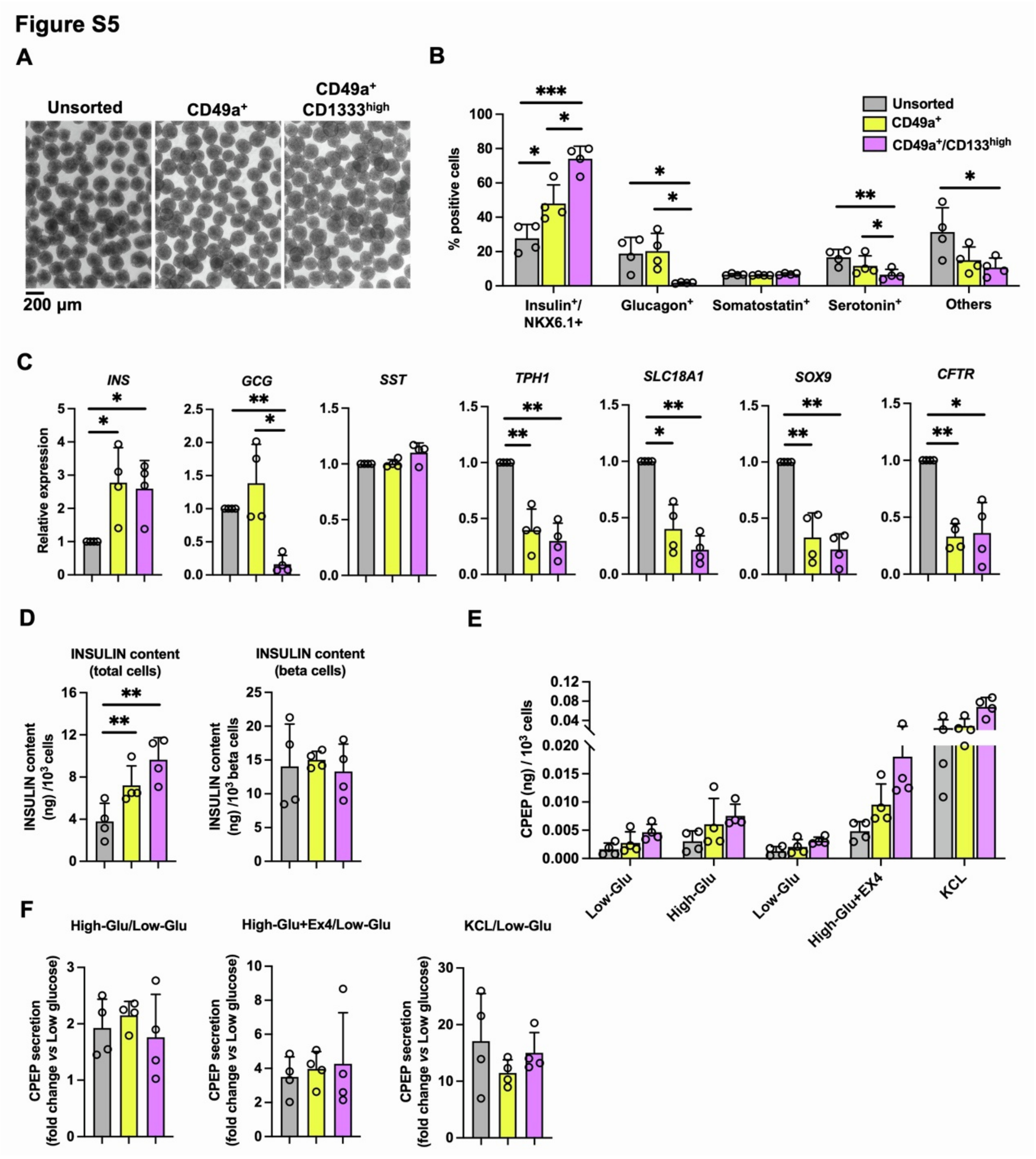

Figure S5. Enhancing the purity of H9 hESC-derived beta cells through CD133 and CD49a-based sorting, related to Figure 3.

(A) Representative bright-field images of unsorted, CD49a-sorted, and CD49a/CD133-sorted cells after 7 days of reaggregation. Scale bar, 200 μm.

(B) Flow cytometry analysis of $INS^{+}NKX6.1^{+}$ beta cells, GCG^{+} alpha/polyhormonal cells, Somatostatin $(SST)^{+}$ delta cells, $Serotonin^{+}$ enterochromaffin cells, and other cells in unsorted, CD49a-sorted and CD49a/CD133-sorted populations. Data are presented as the mean ± SD (n = 4). *$p < 0.05$; **$p < 0.01$; ***$p < 0.001$.

(C) RT-qPCR assessment of marker genes in unsorted, CD49a sorted and CD49a/CD133 sorted populations. Data are presented as the mean ± SD (n = 4). *$p < 0.05$; **$p < 0.01$.

(D) Insulin content measured by ELISA in unsorted, CD49a-sorted, and CD49a/CD133-sorted cell populations. Left: Insulin content normalized by 10^3 total cells; Right: Insulin content

normalized by 10^3 beta cells (total cells / the percentage of beta cells). Data are presented as the mean ± SD (n = 4). $^{**}p < 0.01$.

(E) The C-peptide secretion level after Low glucose (Low-Glu), High glucose (Hi-Glu), Low-Glu, High glucose plus Exendin-4 (Hi-Glu+Ex4) and Low glucose plus KCl (KCl) treatment. Data are presented as mean ± SD (n = 4).

(F) C-peptide secretion stimulation index in static GSIS measured by ELISA in unsorted, CD49a-sorted, and CD49a/CD133-sorted cell populations. Data are presented as the mean ± SD (n = 4).

All the data are from the H9 cell line.

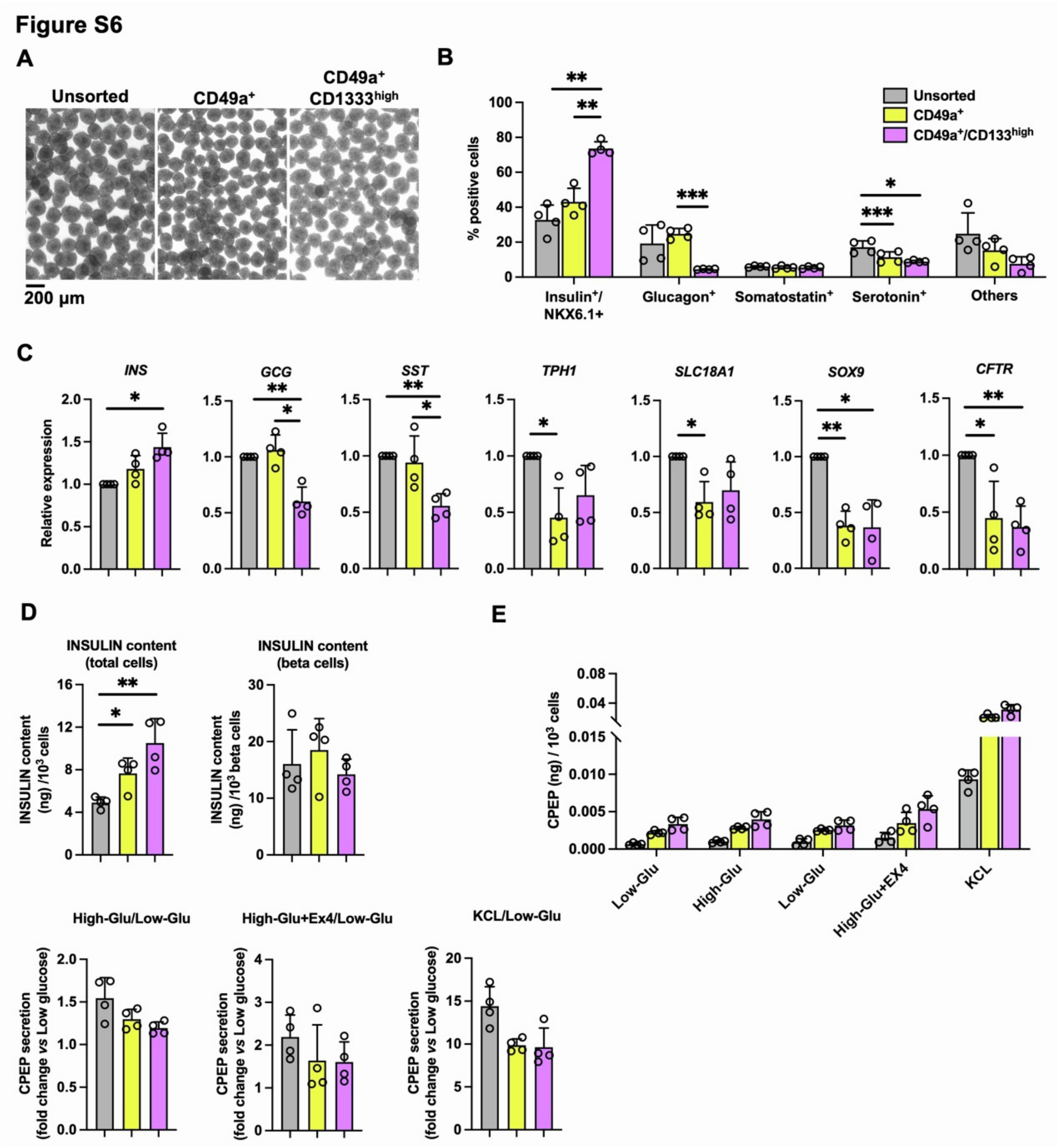

Figure S6. Enhancing the purity of MODY3-iPSC-1-CorB hESC-derived beta cells through CD133 and CD49a-based sorting, related to Figure 3.

(A) Representative bright-field images of unsorted, CD49a-sorted, and CD49a/CD133-sorted cells after 7 days of reaggregation. Scale bar, 200 μm.

(B) Flow cytometry analysis of $INS^+NKX6.1^+$ beta cells, GCG^+ alpha/polyhormonal cells, Somatostatin $(SST)^+$ delta cells, $Serotonin^+$ enterochromaffin cells, and other cells in unsorted, CD49a-sorted and CD49a/CD133-sorted populations. Data are presented as the mean ± SD (n = 4). *$p < 0.05$; **$p < 0.01$; ***$p < 0.001$.

(C) RT-qPCR assessment of marker genes in unsorted, CD49a sorted and CD49a/CD133 sorted populations. Data are presented as the mean ± SD (n = 4). *$p < 0.05$; **$p < 0.01$.

(D) Insulin content measured by ELISA in unsorted, CD49a-sorted, and CD49a/CD133-sorted cell populations. Left: Insulin content normalized by 10^3 total cells; Right: Insulin content

normalized by 10^3 beta cells (total cells / the percentage of beta cells). Data are presented as the mean ± SD (n = 4). $*p < 0.05$; $**p < 0.01$.

(E) The C-peptide secretion level after Low glucose (Low-Glu), High glucose (Hi-Glu), Low-Glu, High glucose plus Exendin-4 (Hi-Glu+Ex4) and Low glucose plus KCl (KCl) treatment. Data are presented as mean ± SD (n = 4).

(F) C-peptide secretion stimulation index in static GSIS measured by ELISA in unsorted, CD49a-sorted, and CD49a/CD133-sorted cell populations. Data are presented as the mean ± SD (n = 4).

All the data are from the MODY3-iPSC-1-CorB cell line.

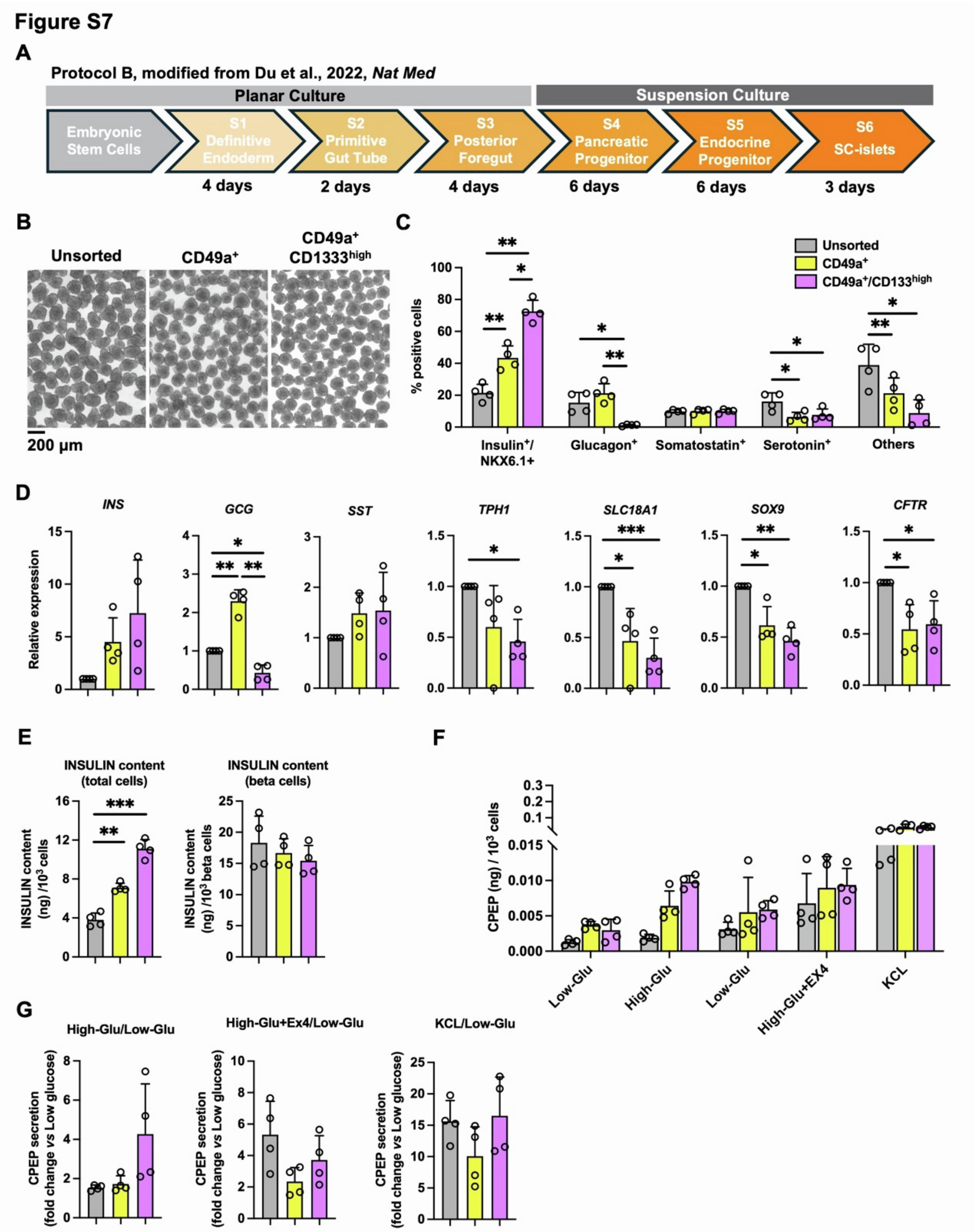

Figure S7. Enhancing the purity of SC-derived beta cells via CD133 and CD49a-based sorting across distinct differentiation protocols, related to Figure 3.

(A) Schematic diagram of a stepwise differentiation protocol to generate SC-islets from hESC. The protocol is modified from Du et al. *Nat Med* (Du et al., 2022).

(B) Representative bright-field images of unsorted, CD49a-sorted, and CD49a/CD133-sorted cells after 7 days of reaggregation. Scale bar, 200 μm.

(C) Flow cytometry analysis of INS^{+}NKX6.1^{+} beta cells, GCG^{+} alpha/polyhormonal cells, Somatostatin (SST)$^{+}$ delta cells, Serotonin^{+} enterochromaffin-like cells, and other cells in

unsorted, CD49a-sorted and CD49a/CD133-sorted populations. Data are presented as the mean ± SD (n = 4). *$p < 0.05$; **$p < 0.01$.

(D) RT-qPCR assessment of marker genes in unsorted, CD49a sorted and CD49a/CD133 sorted populations. Data are presented as the mean ± SD (n = 4). *$p < 0.05$; **$p < 0.01$; ***$p < 0.001$.

(E) Insulin content measured by ELISA in unsorted, CD49a-sorted, and CD49a/CD133-sorted cell populations. Left: Insulin content normalized by 10^3 total cells; Right: Insulin content normalized by 10^3 beta cells (total cells / the percentage of beta cells). Data are presented as the mean ± SD (n = 4). *$p < 0.05$; **$p < 0.01$; ***$p < 0.001$.

(F) The C-peptide secretion level after Low glucose (Low-Glu), High glucose (Hi-Glu), Low-Glu, High glucose plus Exendin-4 (Hi-Glu+Ex4) and Low glucose plus KCl (KCl) treatment. Data are presented as mean ± SD (n = 4).

(G) C-peptide secretion stimulation index in static GSIS measured by ELISA in unsorted, CD49a-sorted, and CD49a/CD133-sorted cell populations. Data are presented as the mean ± SD (n = 4).

All the data are from the H9 cell line.

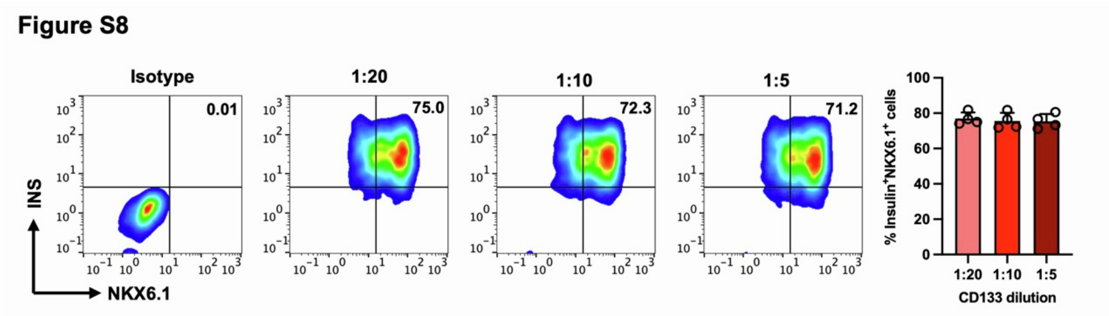

Figure S8. Dilutions of the CD133 antibody from 1:5 to 1:20 yielded comparable beta cell sorting efficiencies, related to Figure 3.

Representative flow cytometry plots (left) and analyses (right) of $INS^{+}NKX6.1^{+}$ beta cells within the $CD49a^{+}$ and $CD133^{high}$ populations using different CD133 antibody dilutions. Data are presented as mean ± SD (n = 4).

Supplemental Experimental Procedures

KEY RESOURCES TABLE

REAGENT or RESOURCE	SOURCE	IDENTIFIER
**Antibodies**		
PE anti-INSULIN	Cell Signaling	#8508<br>RRID: AB_11179076
Alexa fluor 647 anti-INSULIN	BD Biosciences	#565689<br>RRID: AB_2739331
Alexa fluor 488 anti-NKX6.1	BD Biosciences	#563338<br>RRID: AB_2738144
PE anti-NKX6.1	BD Biosciences	#563023<br>RRID: AB_2716792
PE anti-Glucagon	BD Biosciences	#565860<br>RRID: AB_2739382
BV421 anti-Glucagon	BD Biosciences	#565891<br>RRID: AB_2739385
Alexa fluor 647 anti-Somatostatin	BD Biosciences	#570384<br>RRID: AB_3685704
APC anti-Serotonin	Miltenyi Biotec	#130-132-489<br>RRID: AB_2928829
APC anti-CD133	Miltenyi Biotec	#130-113-106<br>RRID: AB_2725935
PE-Vio 770 anti-CD133	Miltenyi Biotec	#130-113-110<br>RRID: AB_2725939
FITC anti-CD49a	Miltenyi Biotec	#130-119-305<br>RRID: AB_2733384
GLUCAGON	Cell Signaling	#8233<br>RRID: AB_10859908
NKX6.1	DSHB	#F55A12<br>RRID: AB_532379
**Chemicals, peptides, and recombinant proteins**		
CHIR99021	Selleck	#S1263
Activin A	PeproTech	#120-14
KGF	PeproTech	#100-19
Vitamin C	Sigma Aldrich	#A4403
LDN193189	Tebu Tech	#04-0074-10
SANT1	Sigma Aldrich	#S4572
Retinoic acid	Sigma Aldrich	#R2625
TBP	Calbiochem	#565740
ALK5iII	Santa Cruz	#sc-221234A
γ-secretase inhibitor XX (γSec-iXX)	Merck Millipore	#565789
SB431542	Selleck	#S1067
Wnt-C59	Selleck	#S7037
EGF	PeproTech	#AF-100-15
ISX9	Selleck	#S7914
Triiodothyronine (T3)	Selleck	#S4217
Zinc sulfate	Sigma	#Z0251
N-Acetyl Cysteine	Sigma	#A9165
Trolox	Merck Millipore	#648471
R428	Med Chem Express	#HY-15150
Bovine serum albumin (BSA)	Sigma Aldrich	#B4287

45 % glucose solution in water	Sigma Aldrich	#G8769
7.5% sodium bicarbonate solution	Thermo Fisher	#25080094
GlutaMAX™ Supplement	Thermo Fisher	#35050061
Penicillin-Streptomycin	Thermo Fisher	#15140122
Heparin	Sigma Aldrich	#H3149-100KU
Y-27632	Merck Millipore	#688000
Biolaminin 521 LN (LN521)	BioLamina	#LN521-05
hESC-Qualified Matrigel	Merck	#CLS354277
Growth factor-reduced Matrigel	Merck	#CLS356231
B27 supplement minus Insulin	Thermo Fisher	#A1895601
B27 supplement	Thermo Fisher	#17504001
Exendin-4	Sigma Aldrich	#E7144
**Critical commercial assays**		
RNeasy Micro Kit	Qiagen	#74004
iScript cDNA Synthesis Kit	Bio-Rad	#1708891
LIVE/DEAD™ Fixable Violet Dead Cell Stain Kit	Thermo Fisher	#L34964
Ultrasensitive C-peptide ELISA	Mercodia	#10-1141-01
Insulin ELISA	Mercodia	#10-1113-10
**Experimental models: Cell lines**		
SA121	Takara bio	RRID: CVCL_B296
H9	WiCell	RRID: CVCL_9773
NEUROG3-GFP	Löf-Öhlin, et al (Lof-Ohlin et al., 2017)	NA
MODY3-iPSC-1-CorB	Hermann, et al (Hermann et al., 2023)	NA
**TaqMan™ probes**		
RPL37A	Thermo Fisher	Hs99999909
INS	Thermo Fisher	Hs02741908
GCG	Thermo Fisher	Hs01031536
SST	Thermo Fisher	Hs00356144
TPH1	Thermo Fisher	Hs00188220
SLC18A1	Thermo Fisher	Hs00915193
SOX9	Thermo Fisher	Hs00165814
CFTR	Thermo Fisher	Hs00357011
MAFA	Thermo Fisher	Hs01651425
UCN3	Thermo Fisher	Hs00846499
**Software and algorithms**		
Fiji 2.0/ImageJ	NIH Image	http://imagej.nih.gov/ij
GraphPad Prism 10	GraphPad	https://www.graphpad.com
FlowJo 10	BD Biosciences	https://www.flowjo.com

hESC differentiation

Protocol A: hPSCs were seeded on growth factor-reduced Matrigel-coated T75 flasks at a density of 1.6×10^5 cells/cm² for pancreatic differentiation. After 24 hours, differentiation toward pancreatic lineages was initiated following a modified protocol based on Rezania et al.(Hermann et al., 2023). On day 13, cells were dissociated and re-aggregated in 6-well suspension plates at 5×10^6 cells per well on an orbital shaker at 100 rpm. On day 20, cells

were sorted using cell surface antibodies, then re-aggregated in AggreWell™ 400 plates (STEMCELL Technologies, #34450) at 1.5 × 10^6 cells per well or Elplasia 96-well round-bottom ultra-low attachment plate (Corning, #4442) at 0.2 × 10^6 cells per well in Stage 6 basal medium. After 24 hours, aggregates were transferred to 6-well suspension plates and cultured for an additional 6 days in Stage 6 basal medium. For in vitro maturation, cells are cultured in Stage 7 medium for 10 days, with medium changed every other day.

Protocol B: hESCs were seeded on LN521-coated T75 flasks at a density of 1.6 × 10^5 cells/cm^2 for pancreatic differentiation. After 24 hours, differentiation toward pancreatic lineages was initiated following a protocol Du et al.(Du et al., 2022) with the following modifications: i) in Stage 1, B27 minus insulin was used in place of standard B27; (ii) on Stage 1, day 1, the medium was devoid of PI103 and Y-27632; (iii) on Stage 1, day 1, the CHIR99021 concentration was reduced from 6 µM to 3 µM; and (iv) on Stage 1, days 2-4, the Activin A concentration was increased from 50 ng/ml to 100 ng/ml. On day 25, cells were sorted using cell surface antibodies, then re-aggregated in AggreWell™ 400 plates (STEMCELL Technologies, #34450) at 1.5 × 10^6 cells per well in Stage 6 basal medium. After 24 hours, aggregates were transferred to 6-well suspension plates and cultured for an additional 6 days in Stage 6 basal medium.

The use of human pluripotent stem cell (hPSC) lines

SA121 (XY, RRID: CVCL_B296), WA09 (H9, XX, RRID: CVCL_9773) and NEUROG3-GFP (XY) hESC lines, and MODY3-iPSC-1-CorB (the corrected MODY3 mutant from the MODY3 patient-derived iPSC line, XX) iPSC line were used in this paper. The SA121 cell line is from Takara Bio (Y00020), the H9 cell line is from WiCell, the NGRUOG3-GFP cell line is derived from SA121 in our previous work(Lof-Ohlin et al., 2017), and the MODY3-iPSC-1-CorB cell line was generated in our previous work(Hermann et al., 2023). The karyotype of hPSCs was normal. For protocol A differentiation: all hPSC lines were cultured on hESC-Qualified Matrigel according to the manufacturer's protocol with daily changes of mTESR™ plus Medium (STEMCELL Technologies, #100-0276) at 37 °C and 5% CO_2. For protocol B differentiation: H9 cell line was cultured on Biolaminin 521 LN (LN521) according to the manufacturer's protocol with daily changes of mTESR™ plus Medium at 37 °C and 5% CO_2. Cells were passaged twice to three times (seeding as 40,000 -60,000 cells/cm^2) weekly at subconfluency using Accutase (Innovative Cell Technologies, #AT-107). The medium was supplemented with 10 µM ROCK inhibitor (Y-27632) on the first day post-passaging. hPSCs were frozen by CryoStor cryopreservation media (Sigma, #C2874). hPSCs were sterile and tested negative for mycoplasma in regular tests. The hPSCs undergo regular checks of the pluripotency by flow cytometry. All experiments were conducted using cells within 2 to 10 passages after thawing.

Flow cytometry analysis

Cells were dissociated by Accutase™. The LIVE/DEAD™ fixable blue dead cell stain kit (Thermo Fisher, #L34964) distinguishes live and dead cells. Then, cells were fixed and stained as previously described(Mamidi et al., 2018) on a Miltenyi MACSQuant analyzer. Antibodies are listed in the Key Resource Table. Data were analyzed using FlowJo software (FlowJo LLC) with established gating strategies. The mean CD133 expression was calculated in FlowJo as the total CD133 signal divided by the number of analyzed cells. Percentile and interquartile range values were calculated using the built-in statistics function in FlowJo.

Immunofluorescence staining

Aggregates were embedded in the hydrogel (Sigma, #TRUE5) on the 8-well chamber (ibidi, # 80827) according to the manufacturer's instructions. Fixation and immunological staining of hESC differentiated cells were performed as previously described(Mamidi *et al.*, 2018). Cells were fixed in 3.7% formalin for 20 minutes, permeabilized with 0.5% Triton X-100 for 60 minutes, and blocked with 5% donkey serum for 2 hours. Primary antibodies are listed in the Key Resource Table. All Alexa Fluor-conjugated secondary antibodies (Thermo Fisher) were used as 1: 500 dilutions. Samples were imaged with Zeiss LSM780 confocal microscopes or the X-light V3 (CrestOptics) spinning disk with the Hamamatsu ORCA-flash camera. Image analyses were performed with Fiji (ImageJ).

RT-qPCR

Total RNA was extracted using the RNeasy Micro Kit. According to the manufacturer's instructions, reverse transcription was performed with iScript cDNA Synthesis Kit. Real-time PCR measurements were performed in technical duplicates using the QuantStudio 7 Flex Real-Time-PCR-System (Thermo Fisher) with TaqMan FAM probes (Thermo Fisher) and TaqMan Master Mix (Thermo Fisher, #4364103). Relative gene expression was determined using the housekeeping genes *RPL37A*. TaqMan probes and primers are listed in the Key Resource Table.

Insulin content and glucose-stimulated insulin secretion (GSIS) assays

On the day of the GSIS assays, 20-40 aggregates were transferred into the low-binding 1.5 ml EP tubes, and then washed twice with Krebs-Ringer bicarbonate buffer (KRB) containing 115 mM NaCl, 5 mM KCl, 1 mM $MgCl_2$, 2.2 mM $CaCl_2$, 20 mM HEPES, 24 mM $NaHCO_3$ and 0.2 % BSA, and pH adjusted to 7.4. All subsequent incubation steps were carried out at 37 °C and 5% CO_2. For GSIS assays, the cells were pre-incubated for 1.5 hrs in KRB supplemented with 1.67 mM glucose before consecutive 30 min incubations in the first KRB with low glucose (2 mM), high glucose (16.67 mM), low glucose (1.67 mM), high glucose plus 10 nM Exendin4 (Ex4) and finally low glucose with KCl (30 mM total). After each step, the medium was

collected, and the cells were washed twice with KRB. Finally, the aggregates were collected, washed once with PBS, and dissociated using Accutase. Half of the cells were used for counting, and the other half were sonicated in H_2O until the solution became clear (4 °C, 20 sec on with 20 sec off). Then, 50 µl of the sonicated solution was transferred into 125 µl 95% HCl-EtOH for insulin content measurement. The remaining sonicated solution was used for genomic DNA quantification. All samples were stored at -80 °C until analysis in technical duplicates with commercially available ELISA kits for Insulin content and secreted human CPEP according to the manufacturer's recommendations.

Reference

Du, Y., Liang, Z., Wang, S., Sun, D., Wang, X., Liew, S.Y., Lu, S., Wu, S., Jiang, Y., Wang, Y., et al. (2022). Human pluripotent stem-cell-derived islets ameliorate diabetes in non-human primates. Nat Med *28*, 272-282. 10.1038/s41591-021-01645-7.

Hermann, F.M., Kjaergaard, M.F., Tian, C., Tiemann, U., Jackson, A., Olsen, L.R., Kraft, M., Carlsson, P.O., Elfving, I.M., Kettunen, J.L.T., et al. (2023). An insulin hypersecretion phenotype precedes pancreatic beta cell failure in MODY3 patient-specific cells. Cell Stem Cell *30*, 38-51 e38. 10.1016/j.stem.2022.12.001.

Lof-Ohlin, Z.M., Nyeng, P., Bechard, M.E., Hess, K., Bankaitis, E., Greiner, T.U., Ameri, J., Wright, C.V., and Semb, H. (2017). EGFR signalling controls cellular fate and pancreatic organogenesis by regulating apicobasal polarity. Nat Cell Biol *19*, 1313-1325. 10.1038/ncb3628.

Mamidi, A., Prawiro, C., Seymour, P.A., de Lichtenberg, K.H., Jackson, A., Serup, P., and Semb, H. (2018). Mechanosignalling via integrins directs fate decisions of pancreatic progenitors. Nature *564*, 114-118. 10.1038/s41586-018-0762-2.
